# Enhancing Binding by Electron Transfer at Heterointerfaces of Biochar‐Modified Hydrogel to Improve Utilization Efficiency of Wastewater Recovered Nutrients

**DOI:** 10.1002/advs.202517709

**Published:** 2026-01-11

**Authors:** Hao Hu, Juncong Zou, Chenglin Zhang, Peng Li, Junnan Li, Yen Wah Tong, Jun Li, Yiliang He

**Affiliations:** ^1^ State Key Laboratory of Green Papermaking and Resource Recycling, School of Environmental Science & Engineering Shanghai Jiao Tong University Shanghai China; ^2^ School of Environmental Science and Engineering Hainan University Haikou Hainan China; ^3^ Department of Chemical and Biomolecular Engineering, 4 Engineering Drive 4 National University of Singapore Singapore Singapore; ^4^ Department of Biomedical Engineering National University of Singapore Singapore Singapore

**Keywords:** biochar, electron transfer, heterointerface, hydrogel, nutrients recovery

## Abstract

Here, this work combines membrane capacitive deionization (MCDI) technology with biochar‐modified hydrogel production to recycle nutrients from wastewater efficiently. MCDI selectively recovered 76.89 ± 5.12% of ammonia and 78.94 ± 3.84% of phosphate from municipal wastewater. The biochar formed layered linear arrays within the hydrogel matrix, and enhanced hydrogen bonding led to the formation of a nanoscale hydrogel coating, creating the distinct heterointerfaces. Interestingly, biochar mediated the enhanced nutrient binding to hydrogel through electron transfer at heterointerfaces, thereby confining the nutrients in nanoscale hydrogel coating and mitigating their release rate. Therefore, the release period of nitrate, ammonia, phosphate, and potassium was prolonged by times of 0.8 to 7.3, 1 to 5.2, 0.1 to 9.1, and 4.7 to 5.7 compared to the unmodified hydrogel. The application of biochar‐modified hydrogel improved soil fertility, which in turn affected the host rhizosphere microbial composition. Furthermore, it resulted in increased lettuce fresh and dry weight compared to fertilization with nutrient‐enriched liquid and the unmodified hydrogel, respectively. This work paves the way towards sustainable nutrient utilization.

## Graphical abstract

Enhancing the utilization efficiency of nutrients recovered from wastewater is achieved through hydrogel modification by biochar. The strategy improves nutrient binding to hydrogels via electron transfer at heterointerfaces, thereby reducing nutrient release rate. Consequently, biochar‐modified hydrogels facilitate vegetable growth by enhancing soil fertility and altering the microbial composition within the rhizosphere of the host.

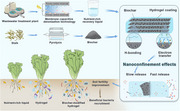

## 1 Introduction

Wastewater is rich in nutrients, and the transformation from mining to resource recovery can be achieved by recycling these nutrients [1]. Nowadays, technologies such as precipitation, adsorption, and ion exchange are employed to recover nitrogen, phosphorus, and potassium, as well as water, from wastewater during treatment [2, 3, 4]. However, the shortcomings of existing technology are likely to contribute to environmental pollution and wastage during the reutilization process [5]. Therefore, the development of novel approaches for the application of recovered resources is significant for improving wastewater resource utilization efficiency. Hydrogels are regarded as promising candidates to replace traditional methods because of their ability to slowly release loaded nutrients while retaining moisture [5, 6, 7, 8]. Hence, the combination of recycling wastewater nutrients with hydrogel synthesis may be conducive to enhancing the resource utilization efficiency, lowering costs, and reducing environmental pollution. However, the practical utilization of hydrogel is limited by certain drawbacks. External factors, such as pH variations, elevated temperatures, and mechanical stress, can disrupt their slow‐release and stability, leading to a rapid cargo delivery [9, 10]. Thus, there is an urgent demand to propose strategies to enhance the performance of hydrogels and thereby improve the utilization efficiency of recovered nutrients.

Biochar, characterized by its porosity, high specific surface area, and electron‐donating and accepting ability, is widely applicable to wastewater treatment, soil amendment, and atmospheric purification [4, 11, 12, 13, 14]. Interestingly, by forming a nanoscale organic coating on its surface, biochar not only immobilizes nutrients but also stimulates microbial activity [15, 16]. Therefore, inspired by this phenomenon and guided by the binary cooperative complementary materials theory, the synthesis of a modified hydrogel with the engineered nanoscale organic coating may present a promising avenue for improving the wastewater recovered nutrients reutilization efficiency. Significantly, since biochar has an electron‐shuttling nature, electron transfer at the biochar‐hydrogel coating heterointerfaces may enhance nutrient loading, thereby confining them at the hydrogel coatings, which may influence nutrient release kinetics [17, 18, 19]. Therefore, it is crucial for wastewater nutrients recycling to comprehend the mechanisms through which biochar influences the physicochemical properties of the composite materials, particularly the interactions between biochar and cross‐linked polymer nanoscale heterointerfaces.

This investigation aims to enhance the utilization efficiency of nutrients recovered from wastewater by modifying hydrogels with biochar through nanoscale heterointerface engineering. Initially, nutrients were selectively extracted from wastewater using membrane capacitive deionization (MCDI) technology, while biochar was produced via pyrolysis of agricultural stalks. Subsequently, we examined the impact of biochar modification on the properties of hydrogels and elucidated the underlying interaction mechanisms. Further, static release experiments were performed to evaluate the release kinetics of nitrogen, phosphorus, and potassium from the biochar‐modified hydrogels. Importantly, a combination of sample characterizations, density functional theory calculations, and finite element analysis was employed to uncover the mechanisms by which biochar modification affects nutrient delivery. Finally, a cultivation trial was conducted to evaluate the efficiency of the modified hydrogel on improving nutrient utilization, thereby establishing a foundation for practical applications. In summary, this work offers novel insights into improving nutrient recycling efficiency by modifying materials, thereby contributing to the development of effective and sustainable technologies for resource‐rich water and wastewater.

## 2 Results and Discussion

### 2.1 Nutrient Recovery From Wastewater by MCDI and Characterization of Stalk‐Derived Biochar

**FIGURE 1:**
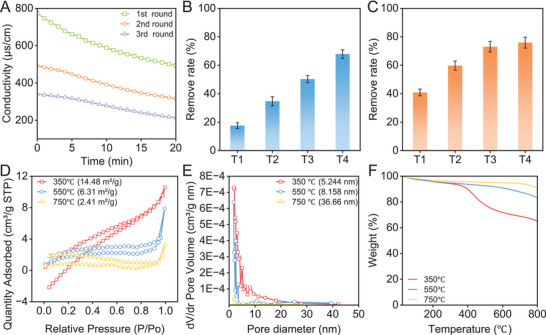
Efficiency of MCDI in recovering nutrients from wastewater and characterization of biochar. (A) The change in conductivity in the feed wastewater during the adsorption processes. (B) Phosphorus removal from the feed wastewater at the end of each adsorption process. (C) Nitrogen removal from the feed wastewater at the end of each adsorption process. (D) Specific surface area of the obtained biochar. (E) Pore volume of the obtained biochar. (F) Thermal degradation curves of the obtained biochar.

Changes in conductivity of the feed solution before and after each adsorption and desorption cycle demonstrated that the MCDI technology functioned effectively for nutrient recovery (Figure 1A). The removal rates of nitrogen and phosphorus from wastewater consistently increased with the number of enrichment cycles due to the selective recovery via electrodes [20, 21]. After four adsorption and desorption trials, the removal efficiencies for phosphorus and nitrogen were recorded at 67.80% and 75.89%, respectively (Figure 1B,C). Correspondingly, the concentrations of phosphorus and nitrogen in the enriched solution were measured at 150.5 mg·L^−1^ and 124.75 mg·L^−1^, respectively. Nutrients were then recovered employing MCDI from municipal wastewater treatment plant A^2^/O process anaerobic tank supernatants that had been subjected to low‐pressure nanofiltration to remove organic matter. The results demonstrated that the NH_4_^+^, NO_3_^−^, PO_4_^3−^, and K^+^ concentrations were 61.10, 7.83, 38.06, and 14.84 mg·L^−1^ in the enriched solution, with a resource recovery of 78.94% for phosphate and 76.89% for ammonia (Table S1). In summary, nitrogen, phosphorus, and potassium can be effectively recovered from wastewater using MCDI technology [21]. Importantly, despite the recovery rate exceeding 70%, additional nutrient supplementation is essential to promote healthy vegetation growth, given the low nutrient concentration in the influent.

Although the specific surface area of the biochar reduced with rising pyrolysis temperature, both dispersion and pore size increased progressively (Figure 1D, E; Figure S1). However, the particle size of biochar was influenced weakly by pyrolysis temperature, without significant differences in median particle size (Figure S2). Higher pyrolysis temperatures resulted in an elevated carbon content in the biochar (Figure 1F; Figure S3). The structural defects of biochar were verified by EPR and Raman spectroscopy. As shown, increasing the pyrolysis temperature initially increased the paramagnetic defects in biochar, followed by a decrease (Figure S4). In contrast, biochar generated at elevated temperatures demonstrated a greater abundance of structural disorder defects and an increase in average crystalline size from 0.49 nm to 0.72 nm (Figure S5) [22]. The differing trends may be attributed to differences in the characterization methods used by the two techniques, as similar results have been observed in previous reports [23, 24]. However, it is important to indicate that the structural disorder of biochar may be more critical to its nutrient slow‐release performance than the paramagnetic defects. Compared to the hydrogel modified by lower temperature pyrolyzed biochar, the higher pyrolysis temperature contributed to the slower nutrient release rate (Figure S6). In addition, the larger pore and defect structure would also benefit the interaction between macromolecules and biochar [25]. Therefore, biochar prepared at 750°C was selected for introduction into the hydrogel to evaluate the effects of the biochar modification on nutrient release from the hydrogel.

### 2.2 Enhanced Hydrogen Bonding Promotes the Formation of Nanoscale Heterointerfaces and Modifies the Hydrogel Properties

**FIGURE 2:**
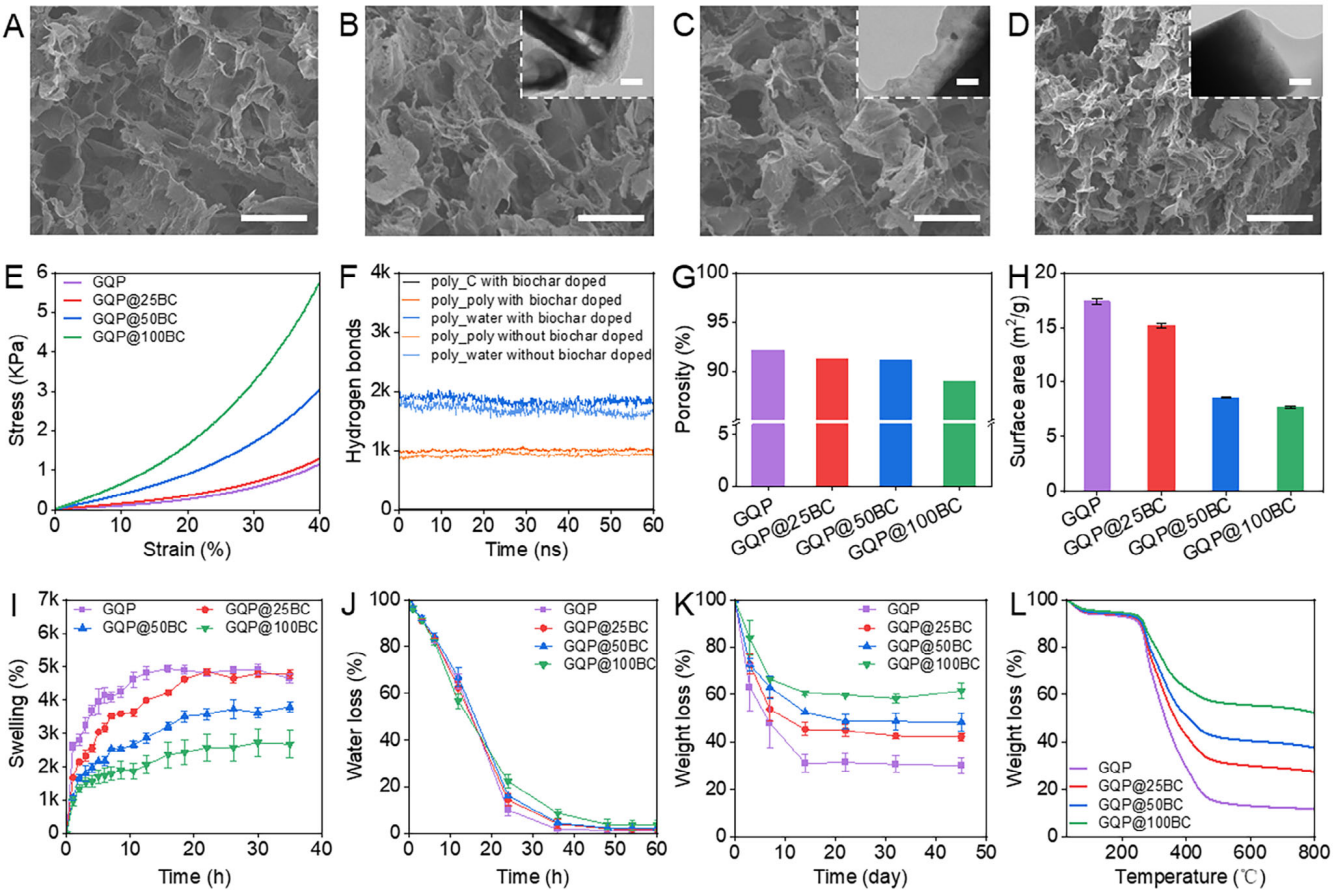
Electron microscope images and performance characterization of the hydrogels. (A–D) SEM images of GQP, GQP@25BC, GQP@50BC, and GQP@100BC hydrogel. The small images in B–D are TEM images of biochar particles encapsulated by the hydrogel coating. (E–L) Stress‐strain curves, number of hydrogen bonds, specific surface area, porosity, swelling performance, water‐holding capacity, biodegradation, and thermal stability of hydrogels. Scale bar: 50 µm for SEM images and 200 nm for TEM images.

Notably, the XRD spectra of biochar‐modified hydrogels only showed faint characteristic peaks of KCl compared to the raw biochar, likely due to the encapsulation of biochar within the crosslinked macromolecular networks, which diminishes peak intensity (Figure S7A). This finding is further supported by alterations in the elemental peak intensities observed in the XPS survey scans and the high‐resolution spectra of individual elements (Figures S7B and S8). Furthermore, FTIR spectra indicated an enhancement of hydrogel‐biochar interactions through hydrogen bonding for GQP@25BC, while higher biochar concentrations led to a redshift of this peak region (Figure S7C). However, it is essential to highlight that the increased gel fractions and the intensities of decomposition peaks suggest a potential strengthening of hydrogen bonding between biochar and the polymer functional groups (Figure S9) [26, 27]. In addition, biochar modification significantly enhanced the light absorption properties of the hydrogel, which gradually increased with rising biochar supplementation, particularly in the visible light region (Figure S10) [28]. The morphology exhibited an increase in roughness with elevated biochar content (Figure 2A–D). Since the powdered biochar was encapsulated within the matrix, visible biochar particles were unobserved on the surface of the hydrogels. Further, as depicted in the inner image of Figure 2B–D, similar to the naturally formed organic coating on the surface of biochar, a hydrogel coating with a thickness of around 200 nm was observed on the surface of the biochar particles [15].

The augmentation of solid content was associated with significant improvements in mechanical properties, evidenced by a more than sixfold increase in compression modulus at the highest biochar concentration compared to the unmodified hydrogel, which may be related to the enhancement of hydrogen bonding (Figure 2E; Figure S11) [17]. Therefore, MD simulations were utilized to determine the number of hydrogen bonds in the GQP and GQP@50BC hydrogel. The results revealed that modifying the hydrogel increased hydrogen bonds at the biochar‐polymer interface by about 13.6% compared to the pure polymer system (Figure 2F). The contribution of direct hydrogen bonding between biochar and polymers was 22.3%, while the polymer‐water hydrogen bonding was enhanced by 12.8% simultaneously. Moreover, the decrease in the system root mean squared deviation (RMSD) value indicated that the amplitude of molecular chain segment dynamics was significantly limited (Figure S12). It is primarily attributed to the dual constraints imposed by the hydrogen bonding on the conformational motion of the polymer chain [29]. Both by fixing the local chain segment conformation through short‐range hydrogen bonding and by inhibiting overall chain slippage via long‐range network synergistic effects [30]. These limitations thus explain why GQP@100BC failed to return to its initial state, as it reduced elasticity while increasing rigidity (Figure S13). This change may be detrimental to fertilization, as soil and root pressure could make it difficult to maintain structure. In addition, soil compaction reduces the spacing between solid particles, resulting in denser pores and increased density, which limits the nutrient diffusion within the substrate [31]. For crops, compacted soil reduces water infiltration, prevents roots from developing naturally, and decreases the ability of plants to uptake nutrients [32]. Consequently, nutrients are confined to the micro‐zone around the hydrogel, decreasing resource utilization efficiency.

The porosity and specific surface area of hydrogels declined with increasing biochar levels, while the pore size remained relatively constant (Figure 2G, H; Figure S14) [33, 34]. This may be attributed to the increased wall thickness and the different cavity structure of hydrogels caused by biochar modification [35, 36]. Meanwhile, due to decreases in porosity and specific surface area, coupled with enhanced hydrogen bonding, modification markedly influenced the swelling and water retention performance of the hydrogels (Figure 2I, J; Figure S15) [37, 38]. Specifically, the swelling capacity decreased by 2.4%, 22.67%, and 44.38% with increasing biochar content, while the water loss percent showed 44.27%, 62.10%, and 122.59% reduction at 24 h compared to the unmodified hydrogel. Lastly, the resistance of modified hydrogels to degradation and thermal decomposition was significantly improved due to the enhanced hydrogen bonding, thereby promoting their long‐term stability (Figure 2K, L). In general, the modification strategy induces enhanced hydrogen bonding interaction between the polymer chains and biochar, which contributes to forming a coating on the biochar surface and creates the nanoscale heterointerfaces. Accordingly, more heterointerfaces formed with increasing biochar dosage, thereby significantly modifying the properties of the hydrogel. These changes provide an opportunity to interact with the mechanisms of nutrient loading and thereby affect the release performance of hydrogels [39, 40].

### 2.3 The Duration of Nutrients Released From Hydrogels is Significantly Prolonged

**FIGURE 3:**
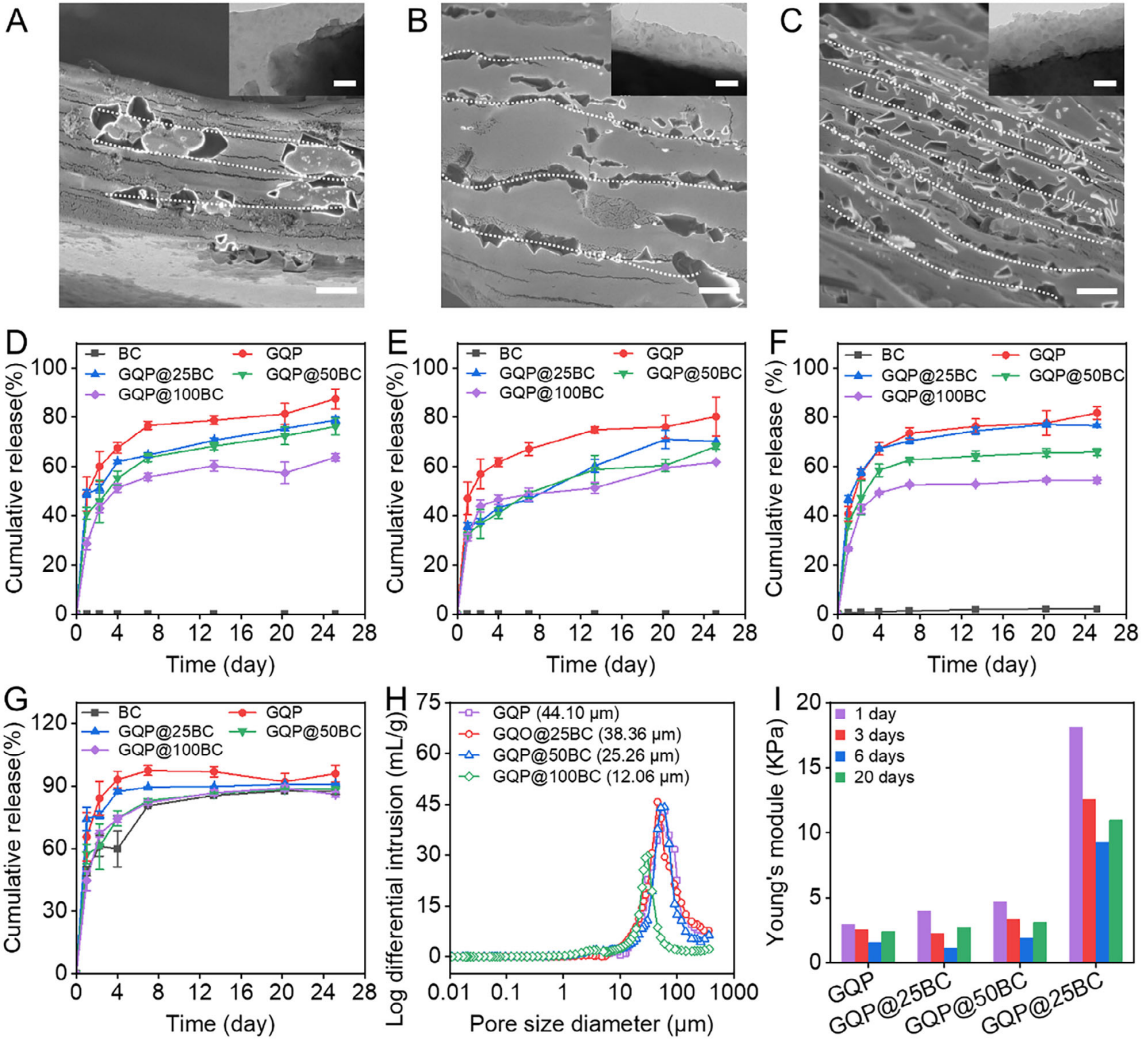
Electron microscope images of biochar‐modified hydrogel, nutrient release kinetics, and physical characterization of hydrogels after nutrients were released. (A–C) SEM images of nutrient‐loaded GQP@25BC, GQP@50BC, and GQP@100BC hydrogel. The inner images are TEM images of biochar particles encapsulated by the hydrogel coating. (D–G) The release kinetics of ammonia, nitrate, phosphate, and potassium. (H) The pore size distribution of hydrogels after nutrients were released. (I) Changes in hydrogel mechanical properties during nutrient release. Scale bar: 1 µm for SEM images and 200 nm for TEM images.

Initially, the physicochemical properties of nutrient‐loaded hydrogels were characterized to evaluate the influence of nutrient incorporation on the hydrogels. Nutrient loading contributed to structural changes in the porosity and specific surface area, which were significantly decreased (Figure S16). Meanwhile, pore size displayed a noticeable decreasing trend with the increased biochar dosage, even though it was increased several‐fold compared to nutrient‐unloaded samples. The SEM images revealed that in the absence of biochar, nutrients were distributed across the hydrogel surface and formed distinct rounded protrusions (Figure S17A). It may be unfavorable to the slow‐release performance of the hydrogel since the surface nutrients are susceptible to rapid release toward lower concentration zones due to the concentration gradients. Conversely, the visible crystalline particles were unobserved on the biochar‐modified hydrogels, and only biochar fragments encapsulated by the organic coating were shown on the surface of the hydrogel matrix (Figure S17B‐D). Therefore, the biochar particles inside the hydrogel were further imaged by SEM. Interestingly, it was demonstrated that the biochar was uniformly and directionally distributed within the hydrogel matrix, presenting a clear linear array (Figure 3A–C). As the biochar dosage increased, the spacing between these arrays decreased, resulting in a higher density of heterointerfaces. Additionally, a hydrogel coating approximately 300 nm thick was observed enveloping the biochar particles, with uniformly sized crystals distributed throughout, indicating a potential enhancement in nutrient loading mediated by biochar. Importantly, an electron‐accumulating region of comparable thickness to the hydrogel coating was also found on the surface of the biochar, suggesting that electron transfer may occur [40, 41].

Static nutrient release trials were conducted to investigate the influence of biochar modification on the nutrient release kinetics of the hydrogels. Initially, an increasing biochar content correlated with a progressive decrease in the quantity of released nutrients over the first three days, indicating a significant influence of biochar on the release rate (Figure 3D–G). Therefore, the time required for nutrients to be released from a series of hydrogels to equivalent levels was markedly extended. Specifically, the release durations for nitrate, ammonia, phosphate, and potassium were prolonged by factors ranging from 0.8 to 7.3, 1 to 5.2, 0.1 to 9.1, and 4.7 to 5.7, respectively, as the biochar content increased from 25 wt% to 100 wt% (Figure S18). The optimal comprehensive slow‐release performance of the GQP@100BC hydrogel was 61.73% for ammonia, 63.69% for nitrate, 54.48% for phosphate, and 85.95% for potassium in 25 days, respectively. Notably, except for potassium, the nutrient release from biochar was nearly negligible, suggesting that the released nitrate, ammonia, and phosphate originated from MCDI sources. Moreover, the nutrient slow‐release performance of the hydrogels reported in this work was significantly higher than that of recently reported hydrogel carriers, demonstrating their great potential for reducing resource input in practical applications (Table S2). Subsequent Korsmeyer‐Peppas kinetic model fitting was utilized to investigate the release mechanism of various nutrients from hydrogels. The fitting results indicate that the *n* value for each nutrient release from different hydrogel materials is smaller than 0.45, which implies that the release behavior of nutrients is governed by diffusion that is driven by concentration gradients (Table S3). Interestingly, the release rate constant exhibited an inverse relationship with biochar levels, highlighting that the presence of heterointerfaces due to biochar modification further enhances the nutrient slow‐release performance of hydrogels.

Following nutrient release, the swelling of the composite materials led to a significant increase in both the porosity and specific surface area of the hydrogels (Figure 3H; Figure S19). However, the influence of modification on the trend in the porous characteristics of hydrogels remained consistent, displaying a reduction with increasing biochar content, suggesting that the intrinsic properties of the hydrogels were largely unaffected after release. Additionally, the mechanical strength of the hydrogels initially declined over the first six days of the release period, and then markedly strengthened by day 20 (Figure 3I; Figure S20). This pattern aligns with the nutrient release profile, characterized by the rapid release stage during the initial six days, followed by a slower release phase. The observed enhancement of hydrogel mechanical properties during the release period is typically attributed to the reorganization of polymer molecular chains, potentially indicating that the released nutrients act as plasticizers [42]. Therefore, following the rapid release phase, the release rate of the residual nutrients significantly decreased, which can be ascribed to an increase in the crosslinking degree of the hydrogel.

**FIGURE 4:**
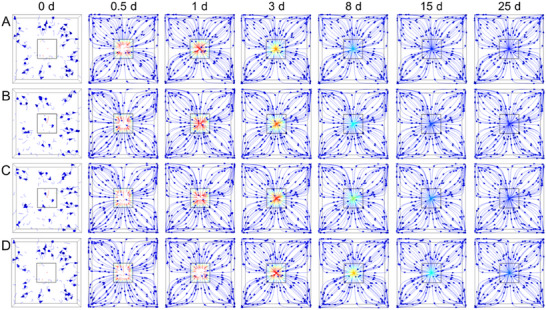
Streamlines of nutrient release from the hydrogel at different predetermined timings derived from finite element simulations. (A) GQP hydrogel. (B) GQP@25BC hydrogel. (C) GQP@50BC hydrogel. (D) GQP@100BC hydrogel.

Nutrient release simulations based on the finite element method demonstrated that the nutrient release kinetics were highly coincident with the experimentally presented results (Figure S21). Although significant differences were nonexistent in nutrient surface release rates between all hydrogels within the first few days of delivery, the increased biochar content resulted in significantly higher release rates than the GQP hydrogel at the later release period, allowing for a more persistent nutrient delivery capability (Figure S22). Moreover, stress simulations during nutrient release indicated that the unmodified hydrogel exhibited faster and more frequent stress changes (Figure S23). The onset of stress change was then delayed for the modified hydrogels, which implies that biochar inhibits the relaxation behavior of the polymer chains due to the reduced distance between biochar lines, thereby prolonging stress maintenance time [17]. Herein, it contributes to the stabilization of the structure and influences the nutrient release rate. Therefore, it allowed the nutrient release rate to display obvious biochar dosage‐dependent behavior (Figure S24). Finally, since the hydrogel swelling behavior was affected by biochar modification, the variation of the nutrient release streamlines revealed that the length inside the hydrogels shortened with the addition of the biochar content at the initiation of release (Figure 4). It was also found that a higher biochar content slowed the prolongation of streamlines within the hydrogels. Herein, modification of the hydrogel with biochar significantly affects the release behavior of loaded nutrients.

### 2.4 Biochar Enhances the Binding Strength Between Nutrients and Hydrogel Matrix via Electron Transfer at Heterointerfaces

According to the nutrient release studies, biochar modification significantly affected the nutrient release duration of hydrogels. Therefore, whether the binding mechanism of nutrients to hydrogels has changed has been investigated. In contrast to the typically crystallized KCl present in biochar, its incorporation as the nutrient in hydrogel demonstrated inhibited crystallization. Consequently, the 2θ value of the (111) of KCl was reduced from 28.2° to 27.4° (Figure S25A). As seen in the FTIR spectra of the hydrogels, distinct vibration regions emerged in the wavenumber range of 1250–1500 cm^−1^ (Figure S25B). Among these are bending vibrations of the hydroxyl group of dihydrogen phosphate in the wavelengths of 1250–1300 cm^−1^, asymmetric stretching vibrations of nitrate in the range of 1350–1450 cm^−1^, and deformation vibrations of ammonia in the range of 1400–1500 cm^−1^ [43]. Concurrently, the strength of the N‐H peak increased significantly owing to the loading of ammonia. Notably, the peak intensity observed in the range of 1350–1450 cm^−1^ suggests that nitrate may bind to the positively charged groups of quaternized chitosan through electrostatic interactions. As biochar levels increased, there was a reduction in peak strength due to the masking effects. This phenomenon implies that biochar facilitates the encapsulation of additional nutrients in the tighter networks, thereby allowing nutrient peaks to persist in the FTIR spectra of modified hydrogels even after release (Figure S26).

**FIGURE 5:**
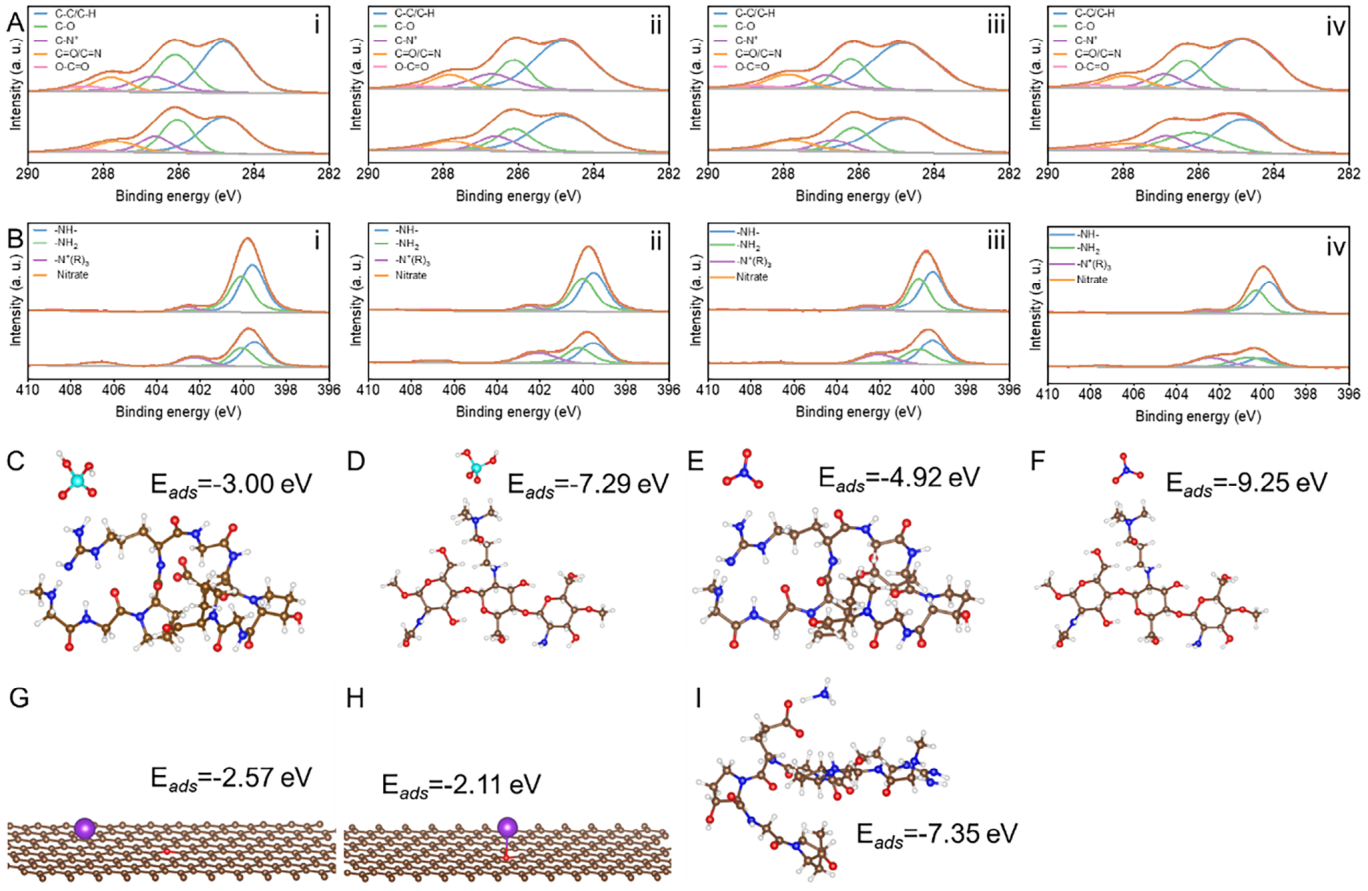
Spectral characterization and DFT calculation of nutrients binding to hydrogels under different conditions. (A) C 1s spectra of hydrogels after nutrients bind to and are released. (B) N 1s spectra of hydrogels after nutrients bind to and are released. Thereinto i, ii, iii, and iv correspond to GQP, GQP@25BC, GQP@50BC, and GQP@100BC hydrogels, respectively. (C) Conformations and binding energy of phosphate in binding to gelatin. (D) Conformations and binding energy of phosphate in binding to quaternized chitosan. (E) Conformations and binding energy of nitrate in binding to gelatin. (F) Conformations and binding energy of nitrate in binding to quaternized chitosan. (G) Conformations and binding energy of the potassium ion in binding to the carbon site of biochar. (H) Conformations and binding energy of the potassium ion in binding to the oxygen site of biochar. (I) Conformations and binding energies of ammonia in binding to gelatin.

To elucidate the specific binding mechanisms of nutrients to hydrogels, XPS analysis and DFT calculations were conducted. Notable alterations in XPS were observed when comparing nutrient‐loaded samples to unloaded counterparts (Figure S27). Specifically, the C 1s spectra revealed a significant reduction in the binding energy of the quaternary ammonium group following nutrient loading, suggesting that electrostatic interactions may govern ion loading due to different charge properties of ions (Figure 5A; Figure S28A, B) [44]. Additionally, a reduction in binding energy was noted in the P 2p high‐resolution spectra after nutrient release, indicating electron loss from phosphate, which corresponded to an increase in binding energy of quaternary ammonium groups when phosphate‐laden (Figures S28C, S29). The change in binding energy of the quaternary ammonium groups was further corroborated by decreased binding energy for quaternary ammonium groups in the N 1s spectra (Figure 5B). DFT calculations reinforced these observations, demonstrating that the energy for quaternary ammonium groups binding to phosphate is lower than that for binding to the amino group of the gelatin, identifying the quaternary ammonium group as the primary binding site (Figure 5C, D). Similarly, binding conformations derived from DFT calculations indicated that, like phosphate, the quaternary ammonium group also serves as the primary binding site for negatively charged nitrate, since the lower adsorption energy is observed between them compared to nitrate and gelatin (Figure 5E, F) [45]. Notably, the absence of changes in K 2p orbitals after potassium ion loading, in conjunction with XRD spectra, suggests that biochar may act as the carrier for potassium (Figures S28D and S30). The findings indicate that potassium ions preferentially associate with the graphite‐like structure of biochar rather than with oxygen atoms (Figure 5G, H) [11]. Furthermore, the negatively charged carboxyl groups of the gelatin, after deprotonation, provide ideal binding sites for positively charged ammonia [46]. It is evidenced by an increase in O‐C = O bond binding energy in the C 1s spectra when ammonia loading and a lower binding energy following its release, suggesting the interaction between them through electrostatic force (Figure 5A, I; Figure S28A).

**FIGURE 6:**
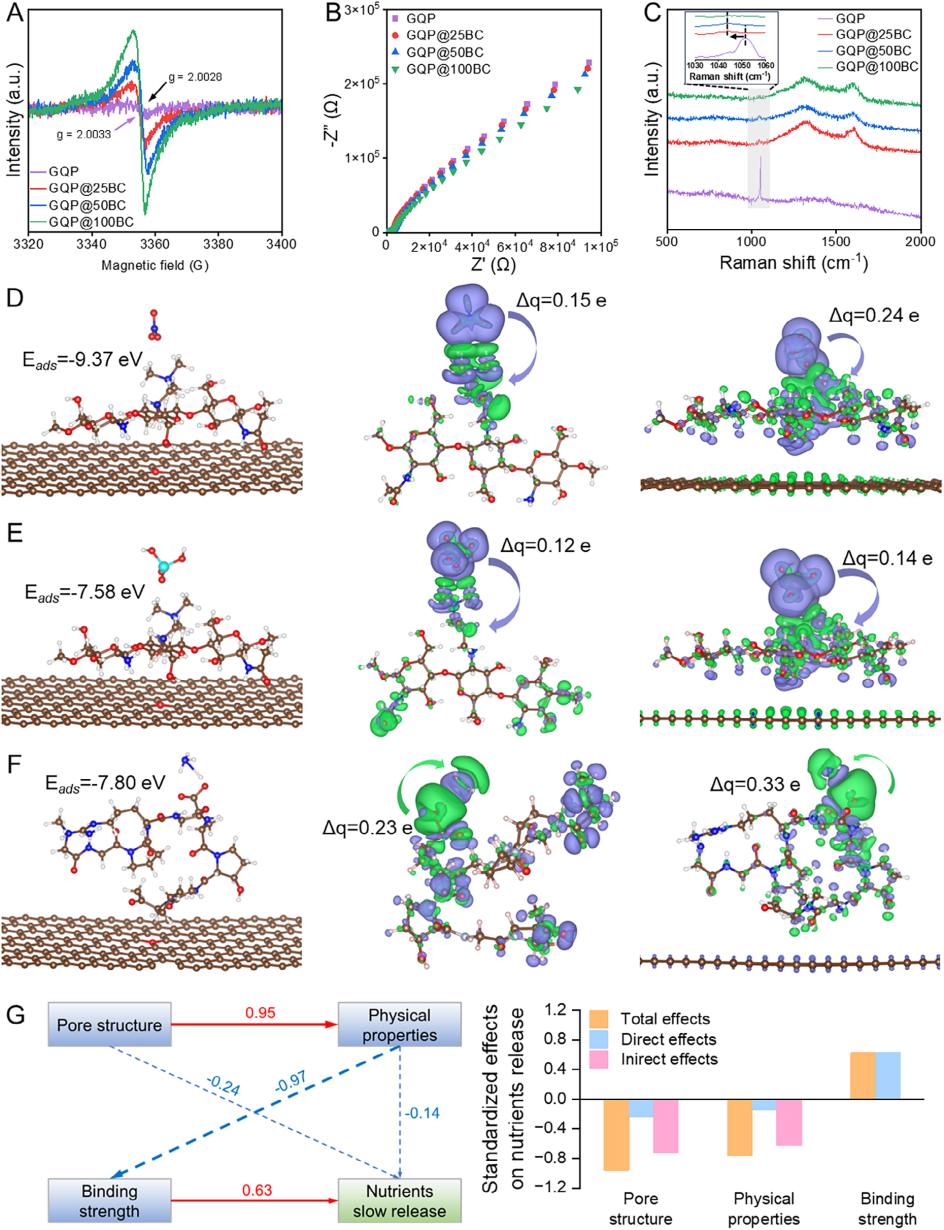
Analysis of biochar modification enhancing nutrient binding to hydrogels. (A) EPR spectra of different hydrogels. The purple arrow indicates the g‐factor value of the GQP hydrogel, while the black arrow represents the g‐factor value of the biochar‐modified hydrogels. (B) Electrochemical impedance spectroscopy Nyquist plots of different hydrogels. (C) Raman spectra of nutrients loaded hydrogels. Except for nitrate ions, other nutrients were undetected, probably because of their low concentrations. (D) The conformations and binding energy of nitrate in binding to hydrogel with biochar modification, and 3D charge density difference for the nitrate binding to quaternized chitosan and biochar‐modified hydrogel. (E) The conformations and binding energy of phosphate in binding to hydrogel with biochar modification, and 3D charge density difference for the phosphate binding to quaternized chitosan and biochar‐modified hydrogel. (F) The conformations and binding energy of ammonia in binding to hydrogel with biochar modification, and 3D charge density difference for the ammonia binding to quaternized chitosan and biochar‐modified hydrogel. (K) Structure equation models illustrate the dominant factor on nutrient release kinetics and standardized total effects.

Given that biochar has the ability to donate and accept electrons, biochar‐mediated electron transfer may exert a decisive influence on extending the nutrient release period from hydrogels after modification. Meanwhile, the electron‐accumulation region on the surface of biochar provides the possibility for electron transfer at heterointerfaces, and therefore, the electrochemical state of hydrogels was characterized [40, 47]. As shown in Figure 6A, the weak signal observed near the g value of 2.0033 resulted from the contribution of unpaired electrons in GQP hydrogel [48]. Obviously, the EPR intensity was enhanced with the increase of biochar content, suggesting that the content of unpaired electrons in hydrogels was increased since the introduction of defective biochar. Herein, the quantity of unpaired spin electrons of hydrogels was quantified, and a significant increase in spin electron number can be observed from 1.43×10^11^ for GQP hydrogel to 2.32×10^12^, 4.21×10^12^, and 6.56×10^12^ for modified hydrogels with biochar increases from 25 wt% to 100 wt% (Figure S31). Moreover, the EPR signal peak of biochar‐modified hydrogels had a slight shift compared with that of GQP hydrogel, which could be attributed to the electron transfer from biochar to macromolecular chains [49]. To further investigate this, electrochemical impedance spectroscopy (EIS) was employed. The Nyquist plots revealed that the low‐frequency impedance decreased with higher biochar dosage, implying a lower charge transfer resistance (Figure 6B) [28, 50].

For further investigation into the influence of electron transfer at heterointerfaces on the binding of nutrients to hydrogels, Raman spectroscopy analysis was performed on the nutrient‐loaded hydrogel materials. On the one hand, the D‐ and G‐band characteristic peaks of biochar were slightly shifted from 1335 cm^−1^ and 1594 cm^−1^ to 1315 cm^−1^ and 1605 cm^−1^ when added into the hydrogel, confirming the strong electronic interactions after modification (Figure 6C) [49]. On the other hand, a very sharp nitrate ν_1_ symmetric stretch band is observed in the Raman spectrum of the GQP hydrogel at 1052 cm^−1^ [51, 52]. Interestingly, after modification by biochar, the characteristic vibrational peak showed a visible red shift to 1042 cm^−1^. Meanwhile, the peak intensity decreased significantly with increasing biochar content, correlating with SEM images showing progressively smoother hydrogel substrate. These findings may indicate that biochar‐mediated electron transfer enhances the interaction between nutrients and functional groups of polymers at the heterointerfaces [53]. Furthermore, the nitrate ν_1_ symmetric stretch band of the biochar‐modified hydrogel still exhibits a red shift compared to the GQP hydrogel during nutrient release (Figure S32). This observation aligns with the increased bonding energy of the various elements evident in the XPS patterns, which correlates with the elevated biochar dosage (Figure S28).

To determine whether the formed heterointerfaces enhance nutrient loading by strengthening biochar‐hydrogel interactions, the binding energy of nutrients and macromolecules in the presence of biochar was calculated. As seen, biochar facilitated binding energy increase and bond length reduction, enhancing nutrient binding to the polymers (Figure 6D–F and Table 1). Given the capacity of biochar for electron donation and acceptance, three‐dimensional differential charge density calculations were performed to elucidate the binding mechanisms of nitrate, phosphate, and ammonia [54]. The observed increase in electron cloud volume in the presence of biochar indicates a notable enhancement in electron transfer (Table 1). Further quantitative insights into electron transfer were derived through charge analysis utilizing the Bader method. The results revealed that the electrons transferred between nitrate and quaternized chitosan, phosphate and quaternized chitosan, as well as ammonia and gelatin, increased from 0.15 e, 0.12 e, and 0.23 e to 0.24 e, 0.14 e, and 0.33 e, respectively (Figure 6D–F). Accordingly, the nanoscale heterointerfaces developed by enhanced hydrogen bonding provide electron transfer venues, promoting tighter nutrient loading by enhanced interfacial binding and confining them within the hydrogel coating, which synergizes with physical diffusion barriers to collectively enable sustained nutrient release [55].

**TABLE 1:** Bond length and electron cloud volume statistics for the primary mode of nutrient binding to hydrogels.

Conformations	Bond length (Å)	Electron cloud volume (Å^3^)
Nitrate and quaternized chitosan	3.74	1310.48
Nitrate and quaternized chitosan with biochar	3.71	3723.34
Phosphate and quaternized chitosan	3.68	1355.37
Phosphate and quaternized chitosan with biochar	3.56	3772.17
Ammonia and gelatin	1.06	1538.52
Ammonia and gelatin with biochar	0.99	3994.09

Based on these characterizations and simulations, we can conclude that the introduction of biochar significantly modified various properties of the hydrogel, including mechanical performance, pore structure characteristics, and nutrient loading mechanisms, ultimately influencing nutrient release kinetics. To elucidate the critical role of these factors, a structure equation model was developed. The results revealed that the binding strength of the ions to the hydrogel emerged as the dominant determinant of the reduced release rate, overshadowing the influence of altered porous structural features or the physical properties of the hydrogel (Figure 6G). This observation highlights the role of the heterostructure‐derived interactions in regulating nutrient release from the hydrogels. Identical results were corroborated through finite element simulations employing a single‐variable control method (Figure S33). In summary, biochar modification created abundant nanoscale heterointerfaces and provided a venue for enhancing the binding strength between the hydrogel and nutrients via electron transfer. This interaction effectively confines the nutrients in the hydrogel coating on the biochar surface, increasing the energy required for their release from the matrix and thereby reducing the release rate [56]. With the increase of biochar dosage, more nanoscale heterointerfaces developed and resulted in more pronounced confinement effects, accompanied by the obstruction of biochar linear biochar arrays, causing the release period for nutrients to reach the same level in different hydrogels to become longer with increased biochar content.

### 2.5 Practical Evaluation of Biochar‐Modified Hydrogel for Enhancing the Recycling Efficiency of Nutrients

**FIGURE 7:**
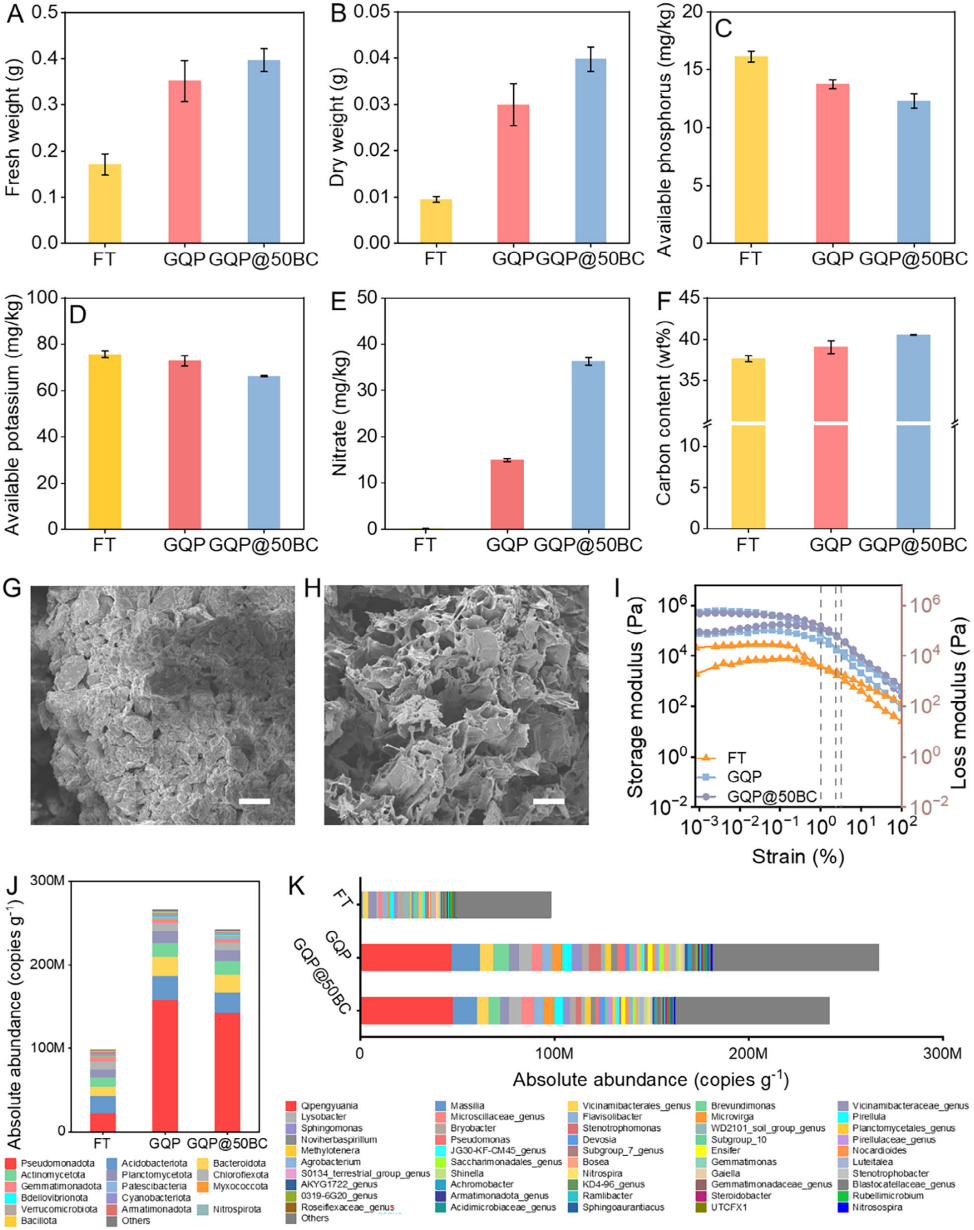
Vegetable growth characteristics and soil physicochemical and ecological functional analysis. (A) The fresh weight of lettuce after the third harvest. (B) The dry weight of lettuce after the third harvest. (C) The available phosphorus level in substrates under different fertilization modes. (D) The available potassium level in substrates under different fertilization modes. (E) The nitrate level in substrates under different fertilization modes. (F) The carbon content in substrates under different fertilization modes. (G) SEM image of GQP hydrogel after the last harvest of lettuce, suggesting the hydrogel was covered with soil particles, and the porous feature has vanished. (H) SEM image of GQP@50BC hydrogel after the last harvest of lettuce, which still retained a distinct porous structure. (I) Rheological performance analysis of soils with different fertilization practices in oscillatory mode. (J) Absolute abundance of rhizosphere bacterial community composition at the phylum level under different fertilization modes. (K) Absolute abundance of rhizosphere bacterial community composition at the genus level (Top 50) under different fertilization modes. Scale bar: 20 µm.

As shown in Figure 7A,B and Figure S34, GQP@50BC exhibited more sustained growth‐promoting effects for vegetables with increasing planting rounds. Compared to FT and GQP hydrogels, the application of GQP@50BC resulted in 132.12% and 12.78% higher vegetable fresh weight and 318.42% and 32.83% higher dry weights, respectively, at the third harvest.

After three rounds of cultivation, although the available potassium and phosphate in the soil decreased, a certain amount remained reserved in the hydrogel, owing to its superior slow‐release performance (Figure 7C, D; Figure S35). Moreover, both ammonia and nitrate levels were significantly higher in the soil when applied with hydrogel rather than recovered nutrient‐rich liquid (Figure 7E; Figure S35). Moreover, following the degradation of hydrogels, their presence in the soil is generally in the small molecule form, which facilitates the increase of the soil organic carbon content (Figure 7F; Figure S35). Simultaneously, biochar itself can enhance soil organic carbon levels. The increased carbon content of the soil is utilized to build the carbon skeleton by vegetable uptake and utilization after being degraded by the microbiome, which might also contribute to the significant increase in the dry weight of the lettuce [7, 12, 57]. It should be noted that gelatin, quaternized chitosan, polyvinyl alcohol, and biochar are biodegradable or biocompatible materials, without adversely influencing soil health when degraded by soil microbes [58, 59, 60]. The previous report indicated that the increased CO_2_ emissions from soil during the degradation of polyvinyl alcohol‐based hydrogels suggest that soil microorganisms can mineralize it [61]. Meanwhile, gelatin and quaternized chitosan are both biopolymeric substances derived from biological sources. In soil, they are initially degraded into smaller molecular fragments such as peptides, amino acids, and glucosamine, and ultimately further broken down by microorganisms into inorganic matters, such as CO_2_, water, and ammonia.

The enhanced hydrogen bonding endowed GQP@50BC with excellent mechanical properties and stability, allowing it to maintain the integrity of the pore structure after long‐term burial (Figure 7G, H). The intact porous structure helps the substrate maintain stable water absorption and retention capacity. Therefore, the soil exhibited a higher storage modulus and shear stress with increasing shear strain (Figure 7I). It implies that it can promote interactions between soil particles and contribute to maintaining the stability of the growing substrate. Furthermore, water infiltration tests suggested that the soil treated with GQP@50BC could store more water (Figure S36). The faster infiltration suggests that the porous nature also provides channels for air and water flow, which enhances the exchange efficiency of water and air considerably.

The findings of the vegetable rhizosphere microbiome absolute quantitative 16S rRNA sequencing demonstrated that fertilization with hydrogels significantly altered the host rhizosphere microbial community composition while decreasing the diversity (Figure S37). Notably, the rhizosphere absolute abundance of bacteria treated with GQP and GQP@50BC showed enhancements of 169.90% and 146.12%, respectively, compared to the FT group (Figure 7J). However, contrary to expectations, the absolute abundance for fertilizing with GQP@50BC was lower than that for the GQP hydrogel. This observation may be attributed to the stress effects of free radicals of biochar on microbial populations [12]. Analysis based on the phylum level demonstrated that although the absolute abundance of several phyla was significantly higher relative to the FT treatment group, the relative abundance was considerably decreased (Tables S4, S5). This phenomenon was linked to the super‐recruitment of *Pseudomonadota*, highlighting its critical role in promoting vegetable growth under the hydrogel application scenario. Further analysis at the genus level revealed that bacteria involved in carbon, nitrogen, phosphorus, and potassium metabolism, including *Qipengyuania*, *Massilia*, *Lysobacter*, *Brevundimonas*, *Ensifer*, *Nitrosospira*, *Pseudomonas*, and *Microvirga*, were significantly enriched in the hydrogel treatment group, both in absolute and relative abundance (Figure 7K; Figure S38) [62, 63, 64, 65, 66, 67]. However, this enrichment is irrelevant to the sustained growth‐promoting ability of the biochar‐modified hydrogel. Investigating the variations of rhizosphere microbes at the genus level suggested that several members, such as *Devosia*, *Pirellulaceae_genus*, and *Saccharimonadales_genus*, exhibited increased absolute (from 5.67×10^6^ to 7.77×10^6^ copies g^−1^) and relative abundances (from 1.28% to 2.01%) under GQP@BC50 fertilization. These genera are responsible for synergistic interactions with mycorrhizae in plant roots and play a role in nitrogen cycling, thereby promoting vegetable growth [68, 69, 70, 71]. Therefore, biochar‐modified hydrogel improved the soil fertility, which fosters the proliferation of beneficial bacteria to modify bacterial composition to enhance the sustainable vegetable growth‐promoting capacity (Figure S39).

## 3 Conclusions

This study presents a novel strategy to enhance the utilization efficiency of nutrients recovered from wastewater. Initially, high‐value nutrients were selectively extracted from actual wastewater using MCDI technology, achieving recovery efficiencies of 76.89 ± 5.12% of ammonia and 78.94 ± 3.84% of phosphate. Subsequently, inspired by the natural organic coatings observed on biochar surfaces, biomass waste‐derived biochar was employed to modify the hydrogel designed for loading and releasing these recovered nutrients. This modification significantly improved the mechanical properties, water retention capacity, and environmental adaptability of the hydrogel. Notably, due to the nanoconfinement effects arising from electron transfer at the heterointerfaces, the nutrient release duration was extended up to more than 5‐fold compared to the unmodified hydrogel. Compared to the 1‐day release period of commercial slow‐release fertilizer, biochar‐modified slow‐release hydrogel fertilizer illustrated much superior release performance [72]. Unfortunately, the cost of biochar‐modified slow‐release hydrogel fertilizer is higher than that of commercial slow‐release fertilizer, as it releases the same amount of nutrients, due to the increase in feedstock cost, operation cost, and capital cost [73]. Nevertheless, with a more extended slow‐release period, hydrogel slow‐release fertilizer offers higher utilization efficiency, reducing both fertilizer input and subsequent labor requirements. Meanwhile, the carbon sequestration ability of biochar is also given to the fertilizer through the incorporation of it into hydrogel. Therefore, compared to commercial slow‐release fertilizers, biochar‐modified slow‐release hydrogel fertilizers present higher usefulness and prospects for application. Consequently, the engineered heterostructure‐modified hydrogel demonstrated a sustainable approach to nutrient delivery. Further, the practical application indicated that the biochar‐modified hydrogel was found to attract beneficial bacteria after soil fertility improved, including *Devosia*, *Pirellulaceae genus*, and *Saccharimonadales genus*, thereby enhancing rhizosphere nutrient cycling and mycorrhizal interactions to promote vegetable growth. Specifically, the fresh and dry weights of lettuce increased by 132.12% and 12.78% as well as 318.42% and 32.83%, respectively, after three rounds of cultivation when fertilized with GQP@50BC compared to FT and GQP. This study offers an efficient, sustainable, and scalable solution for recycling wastewater nutrients, which could enhance wastewater management practices and inspire innovative approaches to resource recycling.

## 4 Methods

### 4.1 Nutrient‐Loaded Hydrogel Synthesis

Hydrogels were manufactured from polyvinyl alcohol, gelatin, quaternized chitosan, and biochar. The polyvinyl alcohol was dissolved by stirring for 1 h at 85°C. When the temperature was decreased to 60°C, gelatin, quaternized chitosan, and biochar were joined and stirred till the polymers were solubilized. Finally, it was cross‐linked with glutaraldehyde to produce the gel solution. The concentration of polyvinyl alcohol, gelatin, and quaternized chitosan was 1.5, 2.0, and 2.0 wt%, respectively. When the mixture solution was sonicated for 10 min in the ice bath to promote the dispersion of biochar and remove the air bubbles, it was repeatedly frozen‐thawed at ‐20°C and ambient to obtain biochar‐modified hydrogels. The hydrogels produced in this study were named GQP, GQP@25BC, GQP@50BC, and GQP@100BC, respectively, and the value indicates the mass fraction of biochar relative to the dry mass of the polymer monomer. The nutrient‐loaded hydrogels were achieved by adding MCDI recovered nutrient‐rich liquid during the manufacturing processes. The recovered nutrient solution has additional nutrients added to meet the nutritional requirements of the vegetation due to the lower nutrient concentration in municipal wastewater.

### 4.2 Nutrient Release Kinetics of Biochar‐Modified Hydrogels

To explore the impacts of biochar modification on the release kinetics of loaded nutrients from the hydrogels, nitrate, ammonia, phosphate, and potassium ions were selected as the subjects. In brief, the hydrogels were immersed in 250 mL of water for 25 days at 25°C. Before analysis, 50 mL of the sample solution was decanted and replenished. Nitrate and ammonia were measured via thymol crystals and Nasher's reagent spectrophotometry, and the others were measured by ICP‐OES. The Korsmeyer‐Peppas kinetic model was applied to fit the release curves of different nutrients for various hydrogels.

### 4.3 Evaluating Biochar Modification on Improving Hydrogel‐Loaded Nutrient Utilization Efficiency

To validate the effects of hydrogel modification by biochar on wastewater recovered resource utilization efficiency enhancement, lettuce was cultivated with three fertilization managements at the same nutrient level: MCDI recovery liquid (FT), nutrient‐loaded unmodified hydrogel (GQP), and nutrient‐loaded biochar‐modified hydrogel (GQP@50BC), respectively. Each pot was filled with 180 g of substrate, made up of 90 g of river sand and 90 g of soil (obtained from a farm in Minhang District, Shanghai, China), to simulate the semi‐arid farmland. Potentially unfavorable effects of river sand on plant growth were removed by washing and sterilizing. Before seeding, saturate each pot with ultrapure water. The developed seedlings were then transplanted to water‐saturated substrates and grown at a temperature of 25/17°C and a day/night cycle of 14/10 for 15 days, with watering every three days during each round. The entire cultivation period consists of three rounds.

### 4.4 Statistical Analyses

The basic data were diagramed in Origin 2024 (OriginLab, USA). Structure equation models (SEMs) were constructed to estimate the direct and indirect effects of factors on nutrient release and vegetable growth based on the maximum likelihood estimation method by AMOS 27 (SPSS Inc., USA). All data were presented as means ± SD and statistically examined by one‐way analysis of variance (ANOVA).

## Conflicts of Interest

The authors declare no conflicts of interest.

## Supporting information




**Supporting File**: advs73710‐sup‐0001‐SuppMat.docx.

## Data Availability

The data that support the findings of this study are available from the corresponding author upon reasonable request.
