# Supplementary material for: Enhancing Binding by Electron Transfer at Heterointerfaces of Biochar‐Modified Hydrogel to Improve Utilization Efficiency of Wastewater Recovered Nutrients
Source: Adv Sci (Weinh). 2026 Jan 11;13(16):e17709. doi: 10.1002/advs.202517709 (PMC13042961; doi:10.1002/advs.202517709)
Supplement: Supplementary file 1 — Supporting File: advs73710‐sup‐0001‐SuppMat.docx. [file ADVS-13-e17709-s001.docx]

Supplementary information

**Enhancing binding by electron transfer at heterointerfaces of biochar-modified hydrogel to improve utilization efficiency of wastewater recovered nutrients**

*Hao Hu*^1^*, Juncong Zou*^2^*, Chenglin Zhang*^1^*, Peng Li*^1^*, Junnan Li*^1^*, Yen Wah Tong*^3^*, Jun Li* ^`^*, Yiliang He*^1*^

1 State Key Laboratory of Green Papermaking and Resource Recycling, School of Environmental Science & Engineering, Shanghai Jiao Tong University, 800 Dongchuan Road, Shanghai 200240, China.

2 School of Environmental Science and Engineering, Hainan University, Haikou, Hainan 570228, China.

3 Department of Chemical and Biomolecular Engineering, National University of Singapore, 4 Engineering Drive 4, Singapore 117585, Singapore.

4 Department of Biomedical Engineering, National University of Singapore, 15 Kent Ridge Crescent, Singapore 119276, Singapore.

* Corresponding author. Email address: [ylhe@sjtu.edu.cn](mailto:ylhe@sjtu.edu.cn)

This Supporting Information includes 57 pages, 39 figures and 6 tables

**Supplementary Text**

**Materials**

Gelatin and polyvinyl alcohol 1799 were purchased from Shanghai Aladdin Biochemical Technology Co., Ltd., China. Quaternized chitosan was purchased from Shanghai Macklin Biochemical Technology Co., Ltd., China. NaH_2_PO_4_, NaNO_3_, NH_4_Cl, and KCl were purchased from Sinopharm Chemical Reagent Co., China. Glutaraldehyde was purchased from Meryer (Shanghai) Biochemical Technology Co., Ltd., China. All the chemical reagents were not further purified before use.

**Preparation of electrodes and recovery of nutrients from wastewater**

A mixture of 5.8 g of activated carbon, 1.5 g of polyvinylidene fluoride, and 1.5 g of graphite powder was dissolved in 30 mL of N-methylpyrrolidone by mechanical stirring for 24 hours. Afterward, the solution was scraped onto the graphite paper by a plate scraper, and then placed in a vacuum drying oven at 80 °C for 3 h to obtain the basic electrode. The modified electrode was obtained by adding 1 g of glutaraldehyde cross-linked polyethyleneimine to the above system.

A cation exchange membrane was attached to the cathode of the electrode when nutrient recovery from wastewater was performed. Firstly, nutrient enrichment was accomplished using 200 mL of simulated wastewater prepared from 1.5 mM (NH_4_)_2_HPO_4_ and 3.5 mM NaCl as the feed solution and 30 mL of ultrapure water as the receiving solution for the desorption processes. The duration of each desorption and adsorption was 20 minutes, and four adsorption and desorption experiments were performed. Following the simulation experiments, a practical study was conducted to recover nitrogen, phosphorus, and potassium from wastewater using the same batch of actual wastewater from a municipal wastewater treatment plant in Shanghai, China, with influent water quality as shown in Table S1.

**Preparation of broomcorn stalk biochar**

The biochar used in this work was obtained by pyrolysis of sorghum stalks, which were obtained from farmland near the university. After eliminating the outer husk, the inner core was washed with water and then dried in an oven. It was then fragmented by a pulverizer and passed through a 100 μm sieve to obtain sorghum straw powder. The biochar was produced by pyrolysis at 350, 550, and 750 ℃ for 2 hours under a nitrogen atmosphere by a tube furnace with a ramp rate of 5 °C min^-1^. After being ground with agate mortar and sieved to yield powdered biochar for future work.

**Characterization**

The morphology of the samples was observed using a scanning electron microscope (SEM, JOEL JSM-6701F, JOEL, Ltd., Tokyo, Japan) and a transmission electron microscope (TEM, JEOL JEM-F200, JOEL, Ltd., Tokyo, Japan). The porosity and pore size of hydrogels were measured by an automatic mercury piezometer (Micromeritics AutoPore V 9620, USA), and the Brunauer-Emmett-Teller (BET) surface area was measured by the automatic surface micropore and physical adsorption instrument at 77 K nitrogen-adsorption method (ASAP 2460, USA).^[1]^ X-ray photoelectron spectroscopy (XPS, Thermo Scientific K-Alpha, USA) was used to analyze the surface element spectrum of hydrogels. Fourier infrared spectroscopy (FTIR, Vertex 70, Bruker; Germany) was used to analyze functional group changes of biochar and hydrogels. The optical properties of the hydrogels were characterized by UV-Vis diffusion reflectance spectroscopy (Shimadzu UV-3600i Plus, Japan) over the wavelength range of 200-800 nm. The crystallographic information was collected on an X-ray diffractometer with a Cu-Kɑ radiation source (XRD-6100, Shimadzu, Japan), and the crystalline size of biochar was calculated by following the Scherrer equation. Room-temperature electron paramagnetic resonance (EPR) was utilized to analyze the vacancy of biochar (Micro ESR, Bruker, Germany), and EPR detected the number of spin electrons for hydrogels under low temperature (100 K) (EMXplus-6/1, Bruker, Germany). The defect structures of biochar and hydrogels were detected by a Raman spectrometer (inVia, Renishaw, UK) with a laser at 532 nm and 785 nm, respectively. The thermal stability was evaluated by the thermogravimetric analyzer (Mettler Toledo, Switzerland) under nitrogen with a 50 mL min^-1^ flow rate, and the temperature was ramped from 25 to 800 ℃ at a heating rate of 10 °C min^-1^. Compression tests of different hydrogels were performed as cylindrical specimens using the Instron mechanical tester (DMA 850, TA, USA). The compression rate was 0.5 mm min^-1^, and stress versus strain curves were obtained. The compressive modulus of the scaffolds was calculated based on the slope of the stress-strain curve at 5-15% of the strain. Electrochemical impedance spectroscopy (EIS) was tested with the frequency range 3000 kHz~10 mHz and alternating current voltage of 5 mV. The gel fraction content, swelling performance, water retention performance, and degradability of hydrogels were investigated following the instructions of a previous report.^[2]^

**DFT Calculation**

The GGA-PBE exchange-correlation function was utilized in calculations to describe the interactions. Set electronic convergence criterion as 1.0$\text{×}$10^-5^ eV, achieved using the Normal (blocked Davidson) algorithm and reciprocal space projection operators, including a vacuum layer of 20 Å to avoid artificial interactions. Non-magnetic calculations were conducted with “normal” precision due to no magnetic moments present in these models, and a default planewave cutoff energy of 400 eV. For sampling integrals over the Brillouin zone, a k-spacing of 0.50 per Å was used, while a finer k-spacing of 0.25 per Å was employed for the precise determination of the electronic density. The binding energy of a matrix on the skeleton surface of hydrogel components was calculated using DFT calculations. The formula used was: E_ads_ = E_tot_ – E_slab_ – E_ab_, where E_slab_, E_ab_, and E_tot_ correspond to the energies of the skeleton surface, the sole matrix, and the total system, respectively. Furthermore, the difference in charge densities was determined using the equation: ∆ρ = ρAB – ρA – ρB, where ρA and ρB are the charge densities of different monomers in a composite, and ρAB is the charge density of the composite.^[3]^ All DFT calculations were performed using MedeA-VASP.

**Numerical simulation**

**Molecular dynamics simulation**

Classic molecular dynamics (MD) simulations were carried out to investigate the hydrogen bonding change of GQP and GQP@50BC hydrogel at the atomic level. For the initial configurations, all the molecules were randomly inserted into a cubic simulation box. The reported force fields were chosen to simulate the hydrogel systems.^[4, 5]^ The molecular force field consists of bonded and nonbonded interactions, and the latter contains van der Waals (vdW) and electrostatic interactions. For each simulation, an energy minimization was first employed to relax the simulation box. Then, an isothermal-isobaric (NPT) ensemble with a 1.0 fs time step is employed to optimize the simulation box, where the temperature is set to 298 K, and the pressure is set to 1.0 atm. The temperature and pressure are kept via the Nose-Hoover thermostat and Parrinello-Rahman barostat, respectively. The NPT optimization time was set to 20.0 ns, which is long enough to obtain a stable box size. Following the NPT simulation, a canonical (NVT) ensemble with 10.0 ns was performed to optimize the simulation box further, and the time step is set to 2.0 fs. At last, another 60.0 ns NPT simulation was performed to collect the trajectory coordinates of molecules. The time step of the NPT simulation was set to 2.0 fs. In all the MD simulations, the motion of atoms was described by classical Newton’s equations, which were solved using the velocity-Verlet algorithm. All simulations were performed using the GROMACS 2018.8 package.^[6]^

**Finite element simulation**

Virtual hydrogel models were created in COMSOL software. The diluted species transport, liquid transfer, and solid mechanics modules built-in COMSOL Multiphysics version 6.1 were utilized to evaluate the impacts of biochar modification on the release kinetics of hydrogels. Four different hydrogel models with specific parameters are detailed in Table S6.

During simulations, a 1 cm^3^ hydrogel model was immersed in the 40 mL bulk solution. The dimensions of the liquid region were 35 mm in length, width, and height. A finely controlled grid was utilized in the simulations to represent mass transfer within the object accurately. The bottom of the constructs and the walls surrounding the medium were designated as having flux-free conditions, thereby preventing any mass transfer at those boundaries. Since the 3D domain has rotational symmetry, one-eighth of the domain could be selected to substitute the entire model for the release simulations. Mass transfer within the porous medium was employed to simulate the water diffusion process and the release of loaded nutrients from the hydrogel. The diffusion process of water into the hydrogel is described by equation (1).

$$\frac{\text{∂cw}}{\text{∂t}}\text{=}\text{∇}\text{∙}\left[ \text{D}\left( \text{cw} \right)\text{∇}\text{cw} \right]\text{ (1)}$$

Where cw and D(cw) are the concentration of water and concentration-dependent diffusion coefficient, respectively. The release of nutrients from the hydrogel to the external solution due to the gradient of concentration difference is described by equations (2) and (3); the initial concentration of the hydrogel was set to C_0_.

$$\frac{\text{∂}\text{c}_{\text{i}}}{\text{∂t}}\text{+}\text{∇}\text{∙}\text{J}_{\text{i}}\text{=}\text{R}_{\text{i}\text{ }}\text{ (2)}$$

$$\text{J}_{\text{i}}\text{=-}\text{D}_{\text{i}}\text{∇}\text{c}_{\text{i}}\text{ (3)}$$

Given the swelling of hydrogels in liquid ambient, the built-in equation (4) for the solid mechanics physical field was applied to simulate this phenomenon during the calculations.

$$\text{ϵ}_{\text{hs}}\text{=}\text{β}_{\text{h}}\text{M}_{\text{m}}\left( \text{c}_{\text{m0}}\text{-}\text{c}_{\text{mo, ref}} \right)\text{ (4)}$$

Where β_h_ is the hygroscopic swelling coefficient of the hydrogels, M_m_ is the molar mass of water, and c_m0_ and c_m0, ref_ are the concentration and reference concentration of water.

The porous mass transfer equations (5) and (6) were applied to the computational process due to the differences in the pore structure features of the hydrogel resulting from biochar modification.

$$\frac{\text{∂(}\text{ϵ}_{\text{p}}\text{c}_{\text{i}}\text{)}}{\text{∂t}}\text{+}\text{∇}\text{∙}\text{J}_{\text{i}}\text{=}\text{R}_{\text{i}}\text{+}\text{S}_{\text{i}}\text{ (5)}$$

$$\text{J}_{\text{i}}\text{=-}\text{D}_{\text{e,i}}\text{∙}\text{∇}\text{c}_{\text{i}}\text{ (6)}$$

Where ϵ_p_ is the porosity of the hydrogel.

**Absolute quantitative sequencing and bioinformatic analysis of bacterial 16S rRNA gene**

To clarify the shift of the rhizosphere microbiome of lettuce after differential fertilization, absolute quantification 16s rRNA amplicon sequencing was conducted with the assistance of the Shanghai G&C Biotechnology Co., Ltd. (China). Briefly, genomic DNA extraction for all samples was performed using the FastDNA® Spin Kit for Soil (MP Biomedicals) according to the manufacturer’s protocol. Gene DNA integrity was tested by agarose gel electrophoresis. DNA concentration was measured using a NanoDrop 2000 spectrophotometer (Thermo Fisher Scientific, Inc.). Nine different spike-in sequences with at least four different concentrations (10^3^, 10^4^, 10^5^, and 10^6^ copies of internal standards) were added to the sample DNA pools. Spike-in sequences consisted of conserved regions identical to those of selected natural 16s rRNA genes and artificial variable regions as mentioned above. Amplification of the V4V5 regions of the 16s rRNA gene was performed to amplify the target fragment.

The raw read sequences were processed in QIIME2. The adaptor and primer sequences were trimmed using the cutadapt plugin. The DADA2 plugin was used for quality control and to identify amplicon sequence variants (ASVs). Taxonomic assignments of ASV representative sequences were performed with a confidence threshold of 0.7 by a rdp-classifier (Version 2.14). Then the spike-in sequences were identified, and reads were counted. A standard curve for each sample was generated based on the read-counts versus spike-in copy number, and the absolute copy number of each ASV in each sample was calculated by using the read-counts of the corresponding ASV. Since the spike-in sequence is not a component of the sample flora, the spike-in sequence needs to be removed in the subsequent analysis. Finally, species annotation of ASV/OTU sequences was performed using QIIME2 software.

**Supplementary Figures**

Figure S1. SEM images of the obtained biochar after pyrolysis at different temperatures. (A) 350 ℃. (B) 550 ℃. (C) 750 ℃. Biochar obtained by pyrolysis at 350 °C showed higher brightness, indicating that it is negatively charged, which may cause difficulty in homogeneous dispersion in the hydrogel if incorporated into the gel.^[7]^ In addition, the biochar prepared at 550 °C also exhibited easier agglomeration compared to the biochar obtained by pyrolysis at 750 °C. Scale bar: 100 μm for (i) and 50 nm for (ii).

Figure S2. Particle size distribution of biochar pyrolyzed at different temperatures.

Figure S3. Spectral characterization of biochar. (A) FTIR spectral characterization. (B) The survey scan of XPS spectral characterization. (C-E) High-resolution XPS spectra of C 1s, O 1s, and N 1s for different biochar. The graph indicates, from bottom to top, biochar obtained at 350 °C, 550 °C, and 750 °C, and the value illustrates the percentage of the element. As shown in the figure, the increase in pyrolysis temperature resulted in an increase in the content of the element C and a decrease in the content of the other element.


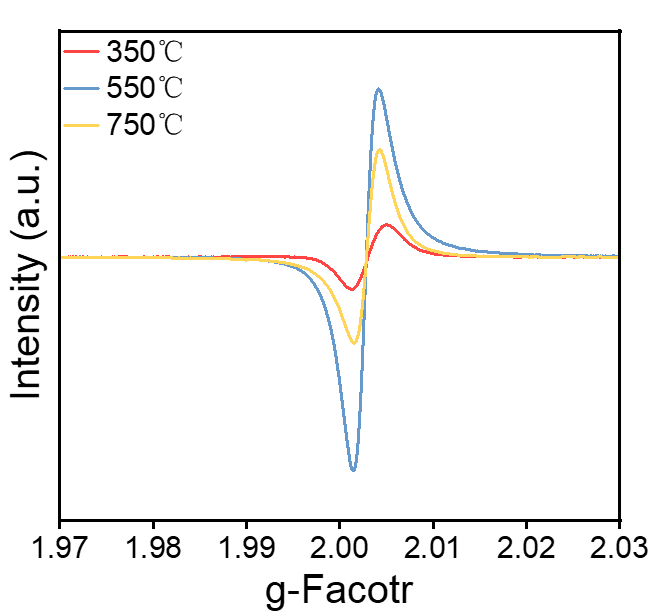


Figure S4. The EPR spectra of biochar particles under different pyrolysis temperatures.

Figure S5. Raman (A) and XRD (B) spectral characterization of biochar produced under different temperatures. This increase in defects was attributed to the intensified condensation of amorphous fragments into graphitic structure by the removal of partial O-containing moieties and corresponded to the intensity decreasing and broadening of the (002) crystal plane of layered graphite carbon on XRD spectra. In addition, the average crystalline size of biochar gradually increases with rising pyrolysis temperature due to the decrease in FWHM of the (100) crystal plane.

Figure S6. The nutrient release kinetics of hydrogels modified by different temperature pyrolyzed biochar. (A) Ammonia. (B) Nitrate. (C) Phosphate. (D) Potassium. Here, the amount of biochar used for the hydrogel modification is 50 %wt of the dry matter mass of the hydrogel.

Figure S7. Spectral characterization of the hydrogels. (A) XRD spectrum of the hydrogels. (B) XPS survey scan spectrum of the hydrogels. (C) FTIR spectrum of the hydrogels.

Figure S8. High-resolution XPS spectra of C 1s, N 1s, K 2p, and P 2p for different hydrogels without nutrient loading. (A) GQP hydrogel. (B) GQP@25BC hydrogel. (B) GQP@50BC hydrogel. (B) GQP@100BC hydrogel.

Figure S9. The gel fraction (A) and differential thermogravimetric curve (B) of hydrogels without nutrient loading. The fitting results suggest that the gel fraction of the hydrogels increases with the amount of biochar. The differential thermogravimetric curves indicate an increase in the pyrolysis peak intensity in the range of 300 to 350 °C caused by biochar modification.


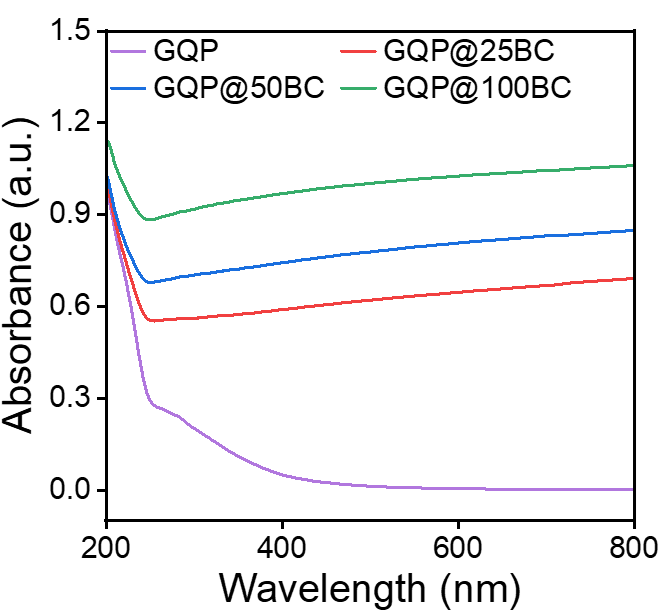


Figure S10. UV–vis absorption spectra of hydrogels.

Figure S11. The compression modulus of hydrogels without nutrient loading.

Figure S12. Root-mean-square deviation of molecular dynamics simulation for GQP and GQP@50BC hydrogel.


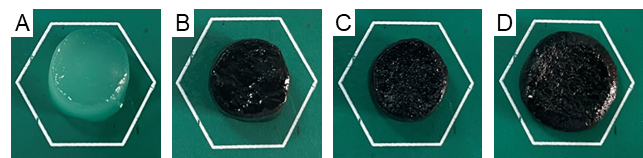


Figure S13. Diagram of the recovery of hydrogels after being compressed. (A) GQP hydrogel. (B) GQP@25BC hydrogel. (B) GQP@50BC hydrogel. (B) GQP@100BC hydrogel.

Figure S14. Pore size distribution of hydrogels without nutrient loading.


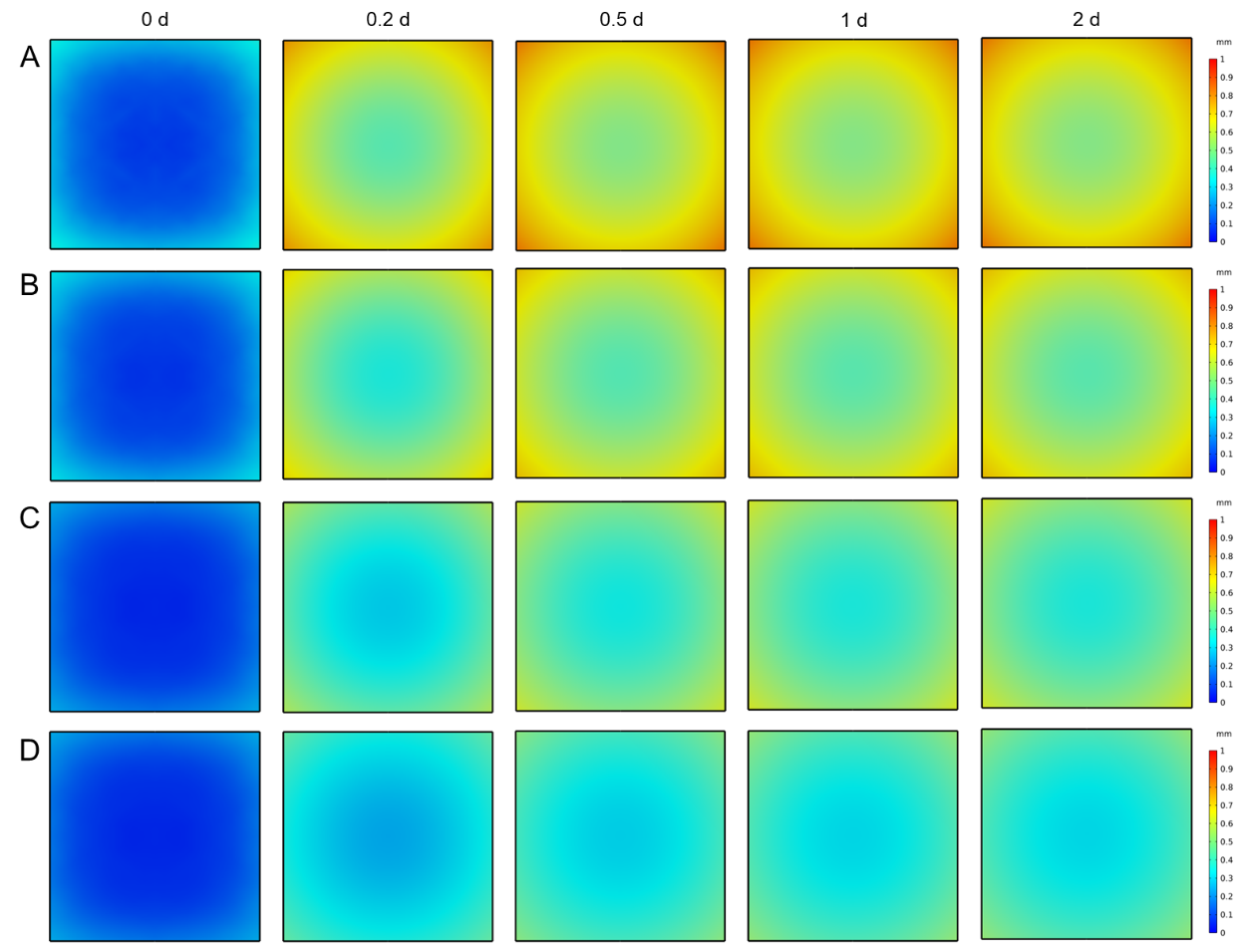


Figure S15. Finite element simulation of the change in hydrogel swelling rate caused by biochar modification.

Figure S16. The specific surface area (A), porosity (B), and pore size distribution (C) of hydrogels with nutrient loading.

Figure S17. SEM images of nutrient-loading hydrogels. (A) GQP hydrogel. (B) GQP@25BC hydrogel. (B) GQP@50BC hydrogel. (B) GQP@100BC hydrogel. Scale bar: 500 μm for (i) and 5 μm for (ii).

Figure S18. Time changes of nutrient release from biochar-modified heterointerface hydrogels compared to GQP hydrogel.

Figure S19. The specific surface area and porosity of hydrogels after nutrient were released.


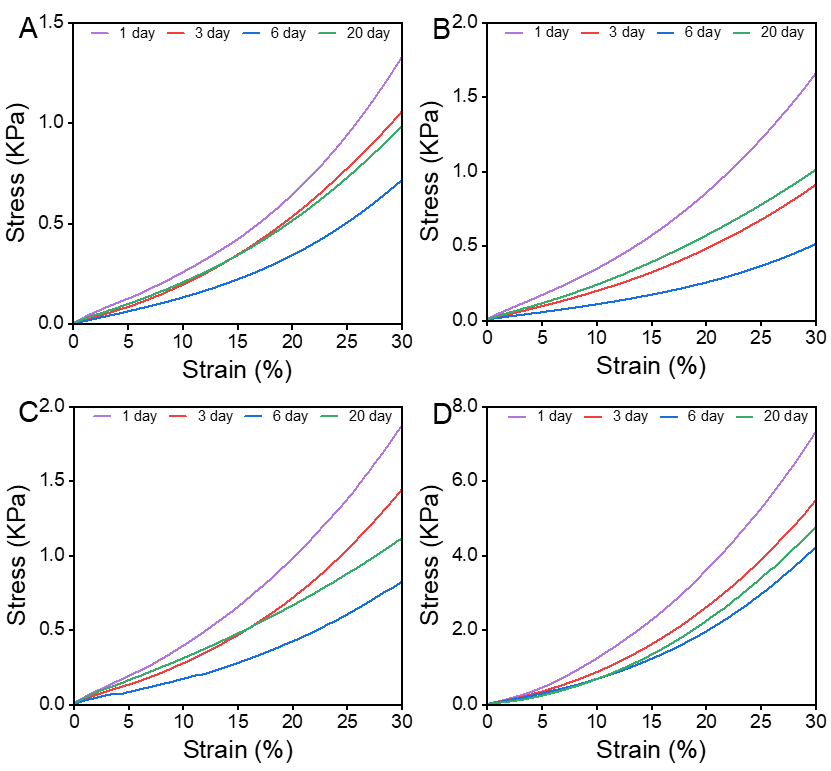


Figure S20. The stress-strain curves of hydrogels after nutrient release. (A) GQP hydrogel. (B) GQP@25BC hydrogel. (B) GQP@50BC hydrogel. (B) GQP@100BC hydrogel.


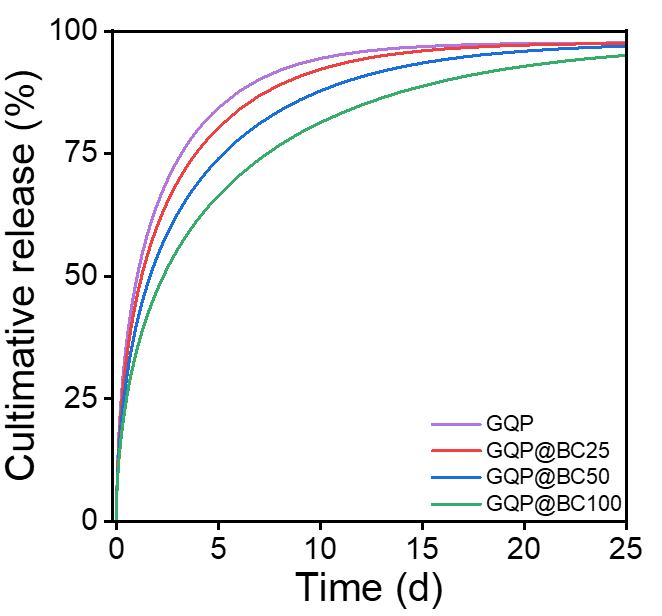


Figure S21.  Simulated cumulative release curves of nutrients from different hydrogels.


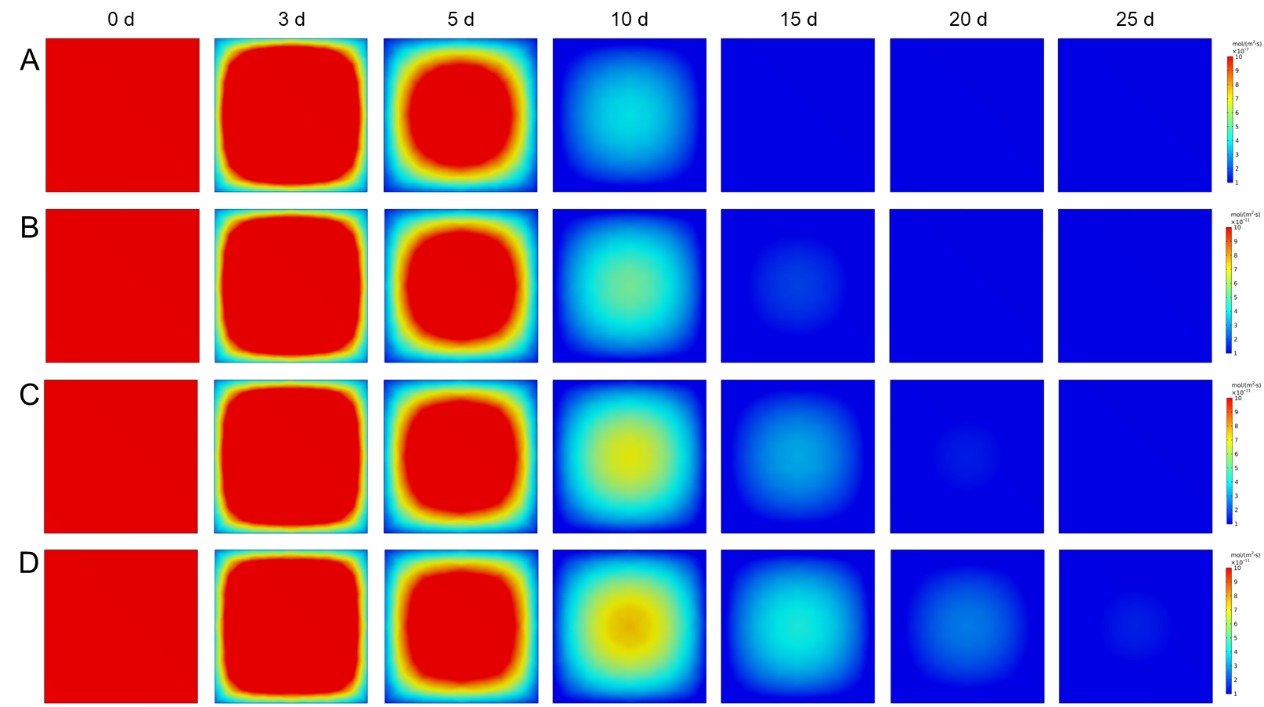


Figure S22. Surface release rates of nutrients from different hydrogels as derived from finite element simulation. (A) GQP hydrogel. (B) GQP@25BC hydrogel. (B) GQP@50BC hydrogel. (B) GQP@100BC hydrogel.


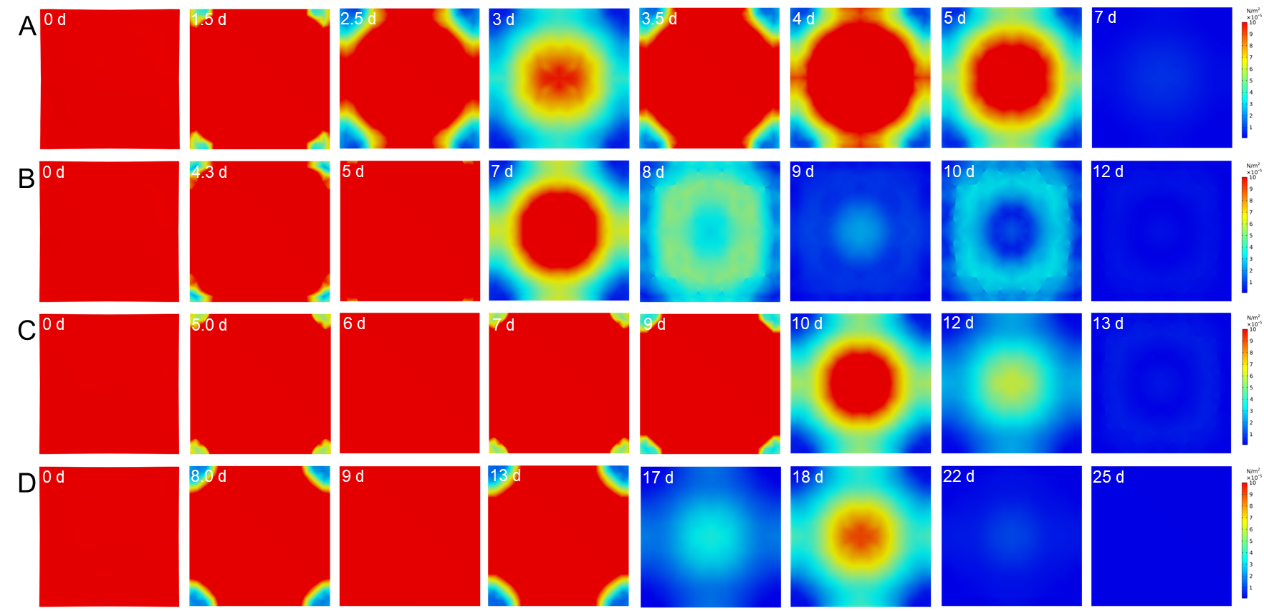


Figure S23. Stress changes of hydrogels during nutrient release. (A) GQP hydrogel. (B) GQP@25BC hydrogel. (B) GQP@50BC hydrogel. (B) GQP@100BC hydrogel.


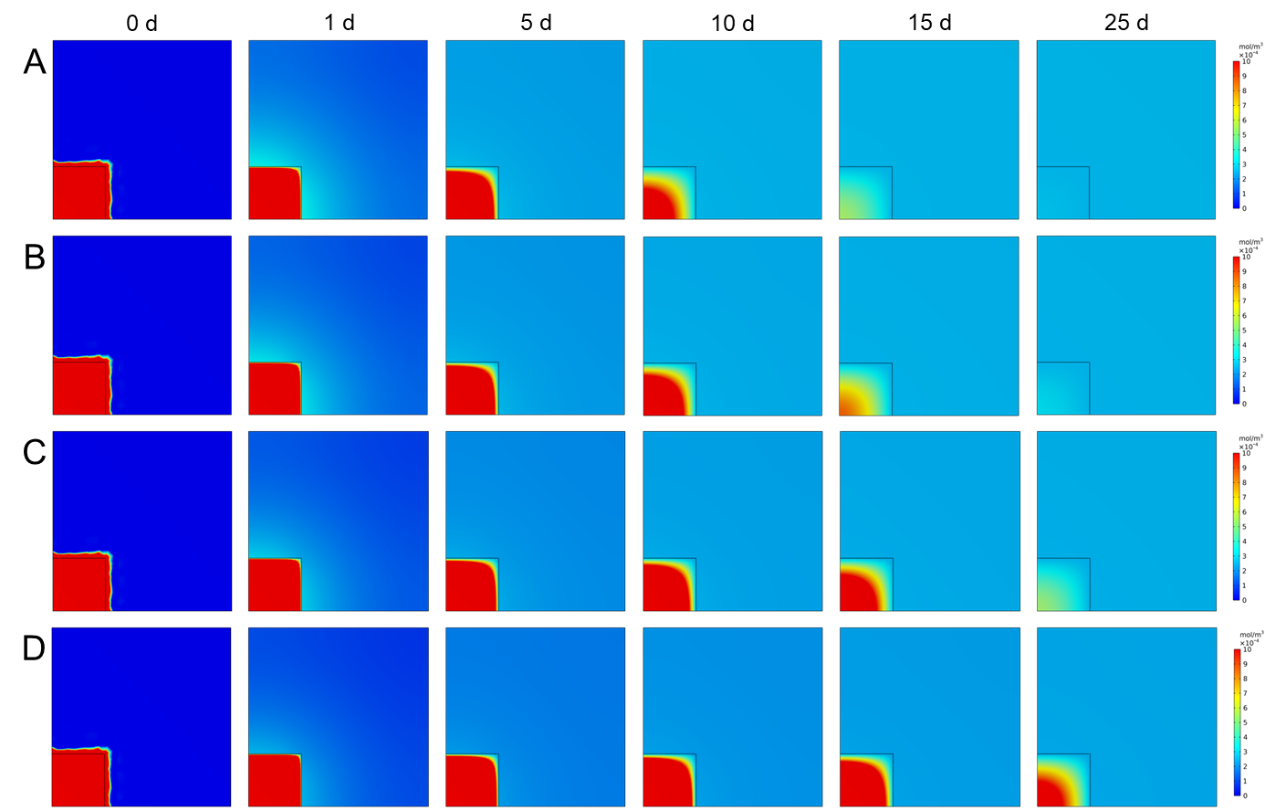


Figure S24. Changes in nutrient concentrations in hydrogels and the liquid phase with time. (A) GQP hydrogel. (B) GQP@25BC hydrogel. (B) GQP@50BC hydrogel. (B) GQP@100BC hydrogel.

Figure S25. The characterization of nutrient-loaded hydrogels. (A) The XRD spectra of hydrogels with nutrient loading. (B) FTIR spectra of hydrogels with nutrient loading.

Figure S26. The FTIR spectra of hydrogels after nutrients release.

Figure S27. XPS survey scan of hydrogels with nutrient loading (A) and after nutrient release (B).

Figure S28. Binding energy shift diagrams of peaks on nutrients unloaded, nutrients loaded, and after nutrients released hydrogels high resolution XPS spectra. (A) C 1s. (B) N 1s. (C) P 2p. (D) K 2p.

Figure S29. High-resolution spectra of the P 2p orbital after loading and release of the phosphate from the hydrogels. (A) GQP hydrogel. (B) GQP@25BC hydrogel. (B) GQP@50BC hydrogel. (B) GQP@100BC hydrogel.

Figure S30. High-resolution spectra of the K 2p orbital after loading and release of the potassium ion from the hydrogels. (A) GQP hydrogel. (B) GQP@25BC hydrogel. (B) GQP@50BC hydrogel. (B) GQP@100BC hydrogel.

Figure S31. Quantitative number of spin electrons of hydrogels.

Figure S32. Raman spectra of hydrogels during nutrient release. (A) The first day. (B) The fifth day. The nitrate ν_1_ symmetric stretch band at Figure A is 1052 cm^-1^ for GQP and 1042 cm^-1^ for GQP@25BC. The nitrate ν_1_ symmetric stretch band at Figure B is 1051 cm^-1^ for GQP and 1045 cm^-1^ for GQP@25BC.

Figure S33. Nutrient release kinetic profiles derived from finite element simulations by controlling the single variable. (A) Different diffusion coefficients of hydrogels. (B) Different modulus of hydrogels. (C) Different hygroscopic coefficient of hydrogels. (D) Different porosity of the hydrogels.

Figure S34. Fresh weight (A and D), dry weight (B and E), and chlorophyll (C and F) content of lettuce harvested at the first and second rounds.

Figure S35. The remaining nutrients in hydrogels (A and B) and other soil nutrient indices (C and D) after three rounds of harvest.

Figure S36. (A) The time required for water to infiltrate the soil with different fertilization modes, completely. (B) Water retention capacity of the substrate under different fertilization modes. The mass of the soil and water is 50 g and 50 g, respectively.

Figure S37. Diversity index and Venn diagram of vegetable rhizosphere bacteria under different fertilization scenarios. (A) Shannon index. (B) Simpson index. (C) PCoA analysis. (D) Venn diagram.

Figure S38. Significance analysis of absolute (A) and relative abundance (B) of vegetable rhizosphere bacteria at the genus level.

Figure S39. Structure equation models illustrate the dominant factor on lettuce growth by applying different fertilization models and standardized total effects. As shown, it first regulates soil fertility conditions by modifying soil moisture, nutrient levels, and soil structural properties when the hydrogel is fertilized. Fertility then contributed to the alteration of the rhizosphere bacterial community composition, which directly affects the growth profile of the vegetables.

**Supplementary Tables**

Table S1. Ion concentration in influent water and recovered liquid before and after MCDI treatment for actual wastewater.

| Ions | Actual wastewater (mg·L^-1^) | Recovered liquid (mg·L^-1^) | Recover rate (%) |
| --- | --- | --- | --- |
| NO_3_^-^ | 1.67 ± 0.06 | 7.83 ± 0.08 | 70.41 ± 3.25 |
| NH_4_^+^ | 11.96 ± 0.99 | 61.10 ± 0.85 | 76.89 ± 5.12 |
| PO_4_^3-^ | 7.24 ± 0.25 | 38.06 ± 0.74 | 78.94 ± 3.84 |
| K^+^ | 16.40 ± 0.16 | 14.84 ± 0.79 | 13.57 ± 0.65 |

Table S2 Comparison of nutrient slow-release performance of various hydrogel carriers over the past five years

| Materials | Nutrients | Release performance | Reference |
| --- | --- | --- | --- |
| Gelatin, cellulose nanocrystal and alginate | NPK fertilizer | 100% for 24 hours in water | ^[8]^ |
| Chitosan and montmorillonite | NPK fertilizer | 55.3% for 15 days in soil | ^[9]^ |
| Wastepaper powder, starch, and itaconic acid | NPK fertilizer | 81% of P, 98% of N and 95% of K for 20 days in water | ^[10]^ |
| Carboxymethyl Starch, polyvinyl alcohol, acrylic acid, and K- montmorillonite | Urea | more than 90% over 10 days | ^[11]^ |
| Hydroxyethyl cellulose, acrylamide | P_2_O_5_, K_2_SO_4_ | Over 95% for 8 days in water | ^[12]^ |
| Chitosan, alginate, and montmorillonite | Phosphate | >99% for 20 days in water | ^[13]^ |
| Leather waste hydrolysate and acrylic acid | Nitrogen | Over 80% for 30 days in water | ^[14]^ |
| Sodium alginate, polyvinyl alcohol, cellulose nanofibers, and | NPK fertilizer | 67.90% of N, 70.78% of P, and 71.12% of K for 30 days in water | ^[15]^ |
| Polyvinyl alcohol, Kaolin, and | Phosphate | 88.6% for 30 days in water | ^[16]^ |
| Chitosan, nano-zeolite, and sago starch | Phosphorus | Approximately 63% for 14 days in water | ^[17]^ |
| Gelatin, polyvinyl alcohol 1799, quaternized chitosan, and biochar | Nitrate, ammonia, phosphate, and potassium | 64% of nitrate, 62% of ammonia, 54% of phosphate, and 86% of potassium for 25 days in water | This work |

| Samples | Index | Ammonia | Nitrate | Phosphate | Potassium |
| --- | --- | --- | --- | --- | --- |
| GQP | R^2^ | 0.98306 | 0.9483 | 0.8816 | 0.6254 |
|  | k | 49.3048 | 52.6690 | 48.7510 | 76.1863 |
|  | n | 0.1525 | 0.1575 | 0.1688 | 0.0836 |
| GQP@25BC | R^2^ | 0.9569 | 0.9760 | 0.9055 | 0.8193 |
|  | k | 31.3821 | 48.0148 | 51.8398 | 75.7674 |
|  | n | 0.2530 | 0.1520 | 0.1344 | 0.0641 |
| GQP@50BC | R^2^ | 0.9819 | 0.97874 | 0.8435 | 0.9146 |
|  | k | 30.8279 | 41.5027 | 42.9252 | 58.8231 |
|  | n | 0.2381 | 0.1909 | 0.1490 | 0.1396 |
| GQP@100BC | R^2^ | 0.9288 | 0.8476 | 0.7470 | 0.8286 |
|  | k | 34.9020 | 36.0680 | 35.3772 | 55.5865 |
|  | n | 0.1734 | 0.1820 | 0.1531 | 0.1588 |

Table S3. Results of Korsmeyer-Peppas kinetic model fitting for the release of different nutrients from hydrogels

Table S4. Absolute quantitatively significant differences in the phylum level of lettuce rhizosphere microbiome under different fertilization practices.

| Phylum | Absolute abundance (copies·g^-1^) | | | P value | | |
| --- | --- | --- | --- | --- | --- | --- |
|  | FT | GQP | GQP@BC50 | GQP vs FT | GQP@BC50 vs FT | GQP@BC50 vs GQP |
| Pseudomonadota | 23129862 | 1.58E+08 | 1.43E+08 | 0.006 | 0.004 | 0.239 |
| Acidobacteriota | 19982498 | 29081336 | 24387303 | 0.006 | 0.002 | 0.043 |
| Bacteroidota | 11495081 | 22074774 | 21383859 | 0.007 | 0.001 | 0.600 |
| Actinomycetota | 11120133 | 16889478 | 16365983 | 0.002 | 0.002 | 0.280 |
| Planctomycetota | 9000127 | 13889792 | 13344961 | 0.002 | 0.001 | 0.239 |
| Chloroflexota | 9670443 | 10221658 | 9037332 | 0.117 | 0.088 | 0.035 |
| Gemmatimonadota | 4464090 | 3924935 | 3724919 | 0.102 | 0.011 | 0.490 |
| Patescibacteria | 311575 | 1507669 | 2779772 | 0.006 | 0.002 | 0.005 |
| Myxococcota | 1693417 | 1127537 | 1018208 | 0.002 | 0.004 | 0.280 |
| Bdellovibrionota | 421852 | 1628639 | 1671078 | 0.002 | 0.006 | 0.638 |
| Cyanobacteriota | 1661969 | 1044966 | 889578 | 0.018 | 0.013 | 0.043 |
| Verrucomicrobiota | 1372234 | 1295714 | 900225 | 0.350 | 0.011 | 0.005 |
| Armatimonadota | 1277671 | 1058373 | 833619 | 0.012 | 0.002 | 0.035 |
| Nitrospirota | 894249 | 1155337 | 925886 | 0.058 | 0.801 | 0.239 |
| Bacillota | 770745 | 677365 | 545594 | 0.102 | 0.013 | 0.073 |

Table S5. Relative quantitatively significant differences in the phylum level of lettuce rhizosphere microbiome under different fertilization practices.

| Phylum | Relative abundance (%) | | | P value | | |
| --- | --- | --- | --- | --- | --- | --- |
|  | FT | GQP | GQP@BC50 | GQP vs FT | GQP@BC50 vs FT | GQP@BC50 vs GQP |
| Pseudomonadota | 23.50251 | 59.63882 | 58.95895 | 5.18E-07 | 0.000792858 | 0.28476114 |
| Acidobacteriota | 20.30444 | 10.94864 | 10.06818 | 0.001181793 | 0.000396819 | 0.28476114 |
| Bacteroidota | 11.68028 | 8.310788 | 8.828227 | 0.000105242 | 0.002134759 | 0.28476114 |
| Actinomycetota | 11.29929 | 6.35861 | 6.75662 | 0.000231143 | 0.000187197 | 0.216846116 |
| Planctomycetota | 9.145128 | 5.229278 | 5.509405 | 3.42E-05 | 0.000306458 | 0.28476114 |
| Chloroflexota | 9.826243 | 3.848286 | 3.73102 | 5.18E-07 | 1.00E-05 | 0.894360374 |
| Gemmatimonadota | 4.536011 | 1.477673 | 1.537815 | 0.000136413 | 0.00044716 | 0.28476114 |
| Patescibacteria | 0.316595 | 0.567613 | 1.147616 | 0.004004254 | 0.0007127 | 0.013784668 |
| Myxococcota | 1.7207 | 0.424499 | 0.420362 | 5.18E-07 | 0.000326473 | 0.851958131 |
| Bdellovibrionota | 0.428648 | 0.613156 | 0.689897 | 0.010378007 | 0.026016501 | 0.28476114 |
| Cyanobacteriota | 1.688745 | 0.393412 | 0.367258 | 0.002096601 | 0.001278503 | 0.614913803 |
| Verrucomicrobiota | 1.394342 | 0.487815 | 0.371654 | 0.001591692 | 0.001083102 | 0.216846116 |
| Armatimonadota | 1.298256 | 0.39846 | 0.344156 | 5.18E-07 | 2.25E-05 | 0.28476114 |
| Nitrospirota | 0.908656 | 0.434965 | 0.382248 | 0.001343159 | 0.000792858 | 0.770119888 |
| Bacillota | 0.783162 | 0.255017 | 0.225246 | 0.000231143 | 0.000326473 | 0.28476114 |

Table S6. Parameters for finite element simulations of nutrient release from hydrogels.

| Parameters | GQP | GQP@25BC | GQP@50BC | GQP@100BC |
| --- | --- | --- | --- | --- |
| Hygroscopic coefficient (m^3^/kg) | 0.001087 | 0.0009661 | 0.0008157 | 0.0007086 |
| Young's modulus (KPa) | 5.867 | 7.02 | 10.05 | 17.81 |
| Diffusion coefficient (m^2^/s) | 9.93×10^-12^ | 7.95×10^-12^ | 5.72×10^-12^ | 3.86×10^-12^ |
| Poisson's ratio | 0.496 | 0.463 | 0.435 | 0.381 |
| Porsity (%) | 91.14 | 90.03 | 88.10 | 84.82 |
| Density (kg/m^3^) | 883.03 | 944.71 | 956.48 | 1024.60 |
| Water diffusion coefficient (m^2^/s) | 3.43×10^-10^ | 2.83×10^-10^ | 2.15×10^-10^ | 1.72×10^-10^ |

Table S7. Results of Korsmeyer-Peppas kinetic model fitting for the release of different nutrients from different biochar-modified hydrogels

| Samples | Index | Ammonia | Nitrate | Phosphate | Potassium |
| --- | --- | --- | --- | --- | --- |
| GQP@BC350 | R^2^ | 0.9962 | 0.9553 | 0.9616 | 0.9543 |
|  | k | 45.4629 | 38.3056 | 57.5900 | 67.0217 |
|  | n | 0.1440 | 0.2283 | 0.1182 | 0.1178 |
| GQP@BC550 | R^2^ | 0.9917 | 0.9800 | 0.9765 | 0.9746 |
|  | k | 34.0578 | 35.6378 | 43.2075 | 63.3027 |
|  | n | 0.2027 | 0.2436 | 0.1697 | 0.1291 |
| GQP@BC750 | R^2^ | 0.9428 | 0.9854 | 0.9280 | 0.9483 |
|  | k | 32.7740 | 38.5637 | 40.1220 | 55.7760 |
|  | n | 0.1896 | 0.2104 | 0.1692 | 0.1538 |

**References**

1. X. Lin; P. Wang; R. Hong; X. Zhu; Y. Liu; X. Pan; X. Qiu; Y. Qin, *Advanced Functional Materials* **2022,** *32*, 2209262.

2. H. Hu; P. Li; Y. W. Tong; J. Li; Y. He, *Chem. Eng. J.* **2024,** *499*, 155943.

3. J. Zou; S. Wu; Y. Lin; X. Li; Q. Niu; S. He; C. Yang, *Environ. Sci. Technol.* **2024,** *58*, 14895-14905.

4. M. Holmboe; P. Larsson; J. Anwar; C. A. S. Bergström, *Langmuir* **2016,** *32*, 12732-12740.

5. Y. Tang; Z. Yu; L.-h. Tam; A. Zhou; D. M. Li, *Mater. Today Commun.* **2022,** *33*, 104834.

6. B. Hess; C. Kutzner; D. van der Spoel; E. Lindahl, *J. Chem. Theory Comput.* **2008,** *4*, 435-447.

7. R. Scipioni; P. S. Jørgensen; D.-T. Ngo; S. B. Simonsen; Z. Liu; K. J. Yakal-Kremski; H. Wang; J. Hjelm; P. Norby; S. A. Barnett; S. H. Jensen, *Journal of Power Sources* **2016,** *307*, 259-269.

8. D. M. do Nascimento; Y. L. Nunes; J. P. A. Feitosa; A. Dufresne; M. d. F. Rosa, *International Journal of Biological Macromolecules* **2022,** *216*, 24-31.

9. Z. Dou; M. V. Bini Farias; W. Chen; D. He; Y. Hu; X. Xie, *Frontiers of Environmental Science & Engineering* **2022,** *17*, 53.

10. A. Bora; D. Sarmah; N. Karak, *International Journal of Biological Macromolecules* **2023,** *253*, 126555.

11. A. El Idrissi; F. Tayi; O. Dardari; A. Akil; O. Amadine; L. Lu; M. Zahouily; Y. Essamlali, *ACS Sustainable Chemistry & Engineering* **2025,** *13*, 7286-7298.

12. M. M. Ghobashy; M. A. Amin; M. A. Ismail; A. I. Nowwar; M. A. El-diehy; H. M. Gayed, *International Journal of Biological Macromolecules* **2023,** *252*, 126467.

13. C. Cao; T. Huo; P. Liu; J. Long; Y. Ma; S. I. Jahan; T. T. Manjoro; F. Dong, *International Journal of Biological Macromolecules* **2025,** *305*, 141276.

14. Z.-Y. Hu; G. Chen; S.-H. Yi; Y. Wang; Q. Liu; R. Wang, *Journal of Environmental Chemical Engineering* **2021,** *9*, 106146.

15. S. Liu; Q. Wu; X. Sun; Y. Yue; B. Tubana; R. Yang; H. N. Cheng, *International Journal of Biological Macromolecules* **2021,** *172*, 330-340.

16. N. Sharma; A. Singh; R. K. Dutta, *Polymer Bulletin* **2021,** *78*, 2933-2950.

17. R. Pimsen; P. Porrawatkul; P. Nuengmatcha; S. Ramasoot; S. Chanthai, *Journal of Coatings Technology and Research* **2021,** *18*, 1321-1332.
